# Mitochondrial genes support a common origin of rodent malaria parasites and Plasmodium falciparum's relatives infecting great apes

**DOI:** 10.1186/1471-2148-11-70

**Published:** 2011-03-15

**Authors:** Samuel Blanquart, Olivier Gascuel

**Affiliations:** 1 Méthodes et Algorithmes pour la Bioinformatique, LIRMM, UMR 5506, CNRS-Université de Montpellier 2, 161 rue Ada, 34392 Montpellier Cedex 5, France; 2 Goldman Group, European Bioinformatics Institute, Wellcome Trust Genome Campus, Hinxton, Cambridge CB10 1SD, UK; 3 Equipe Bonsai, Institut National de Recherche en Informatique et en Automatique, INRIA Lille Nord Europe, 40 avenue Halley, 59650 Villeneuve d'Ascq, France

## Abstract

**Background:**

*Plasmodium falciparum *is responsible for the most acute form of human malaria. Most recent studies demonstrate that it belongs to a monophyletic lineage specialized in the infection of great ape hosts. Several other *Plasmodium *species cause human malaria. They all belong to another distinct lineage of parasites which infect a wider range of primate species. All known mammalian malaria parasites appear to be monophyletic. Their clade includes the two previous distinct lineages of parasites of primates and great apes, one lineage of rodent parasites, and presumably *Hepatocystis *species. *Plasmodium falciparum *and great ape parasites are commonly thought to be the sister-group of all other mammal-infecting malaria parasites. However, some studies supported contradictory origins and found parasites of great apes to be closer to those of rodents, or to those of other primates.

**Results:**

To distinguish between these mutually exclusive hypotheses on the origin of *Plasmodium falciparum *and its great ape infecting relatives, we performed a comprehensive phylogenetic analysis based on a data set of three mitochondrial genes from 33 to 84 malaria parasites. We showed that malarial mitochondrial genes have evolved slowly and are compositionally homogeneous. We estimated their phylogenetic relationships using Bayesian and maximum-likelihood methods. Inferred trees were checked for their robustness to the (i) site selection, (ii) assumptions of various probabilistic models, and (iii) taxon sampling. Our results robustly support a common ancestry of rodent parasites and *Plasmodium falciparum's *relatives infecting great apes.

**Conclusions:**

Our results refute the most common view of the origin of great ape malaria parasites, and instead demonstrate the robustness of a less well-established phylogenetic hypothesis, under which *Plasmodium falciparum *and its relatives infecting great apes are closely related to rodent parasites. This study sheds light on the evolutionary history of *Plasmodium falciparum*, a major issue for human health.

## Background

Malaria is an overwhelming public health problem all over the world. It kills one to three million people annually and infects 200 to 500 million others [1]. Human malaria is induced by infections caused by a range of eukaryotic protists belonging to the phylum *Apicomplexa*. These organisms possess an endosymbiont of red algal origin [2] derived into an apical organelle, the apicoplast. This organelle is specialized in host cell invasion [3]. Within *Apicomplexa*, malaria parasites, also called *Haemosporidia*, are characterized by their infection of vertebrate hosts, haemoglobin digestion, and a complex life cycle involving dipteran vectors feeding from their vertebrate hosts' blood [4].

*Haemosporidia *include the genera *Leucocytozoon *(bird parasites), *Haemoproteus *and *Parahaemoproteus *(Sauria, *i.e*. bird and reptile parasites), *Plasmodium *(saurian and mammalian parasites) and *Hepatocystis *(mammalian parasites) [4-6]. These five genera have long been defined by their morphological differences (e.g. storage of products of haemoglobin degradation in the case of *Haemoproteus*, *Parahaemoproteus*, *Plasmodium *and *Hepatocystis*), variations in their life cycle (e.g. asexual replication stage in erythrocytes for *Haemoproteus*, *Parahaemoproteus *and *Plasmodium*) and host and vector specificity [4,5,7,8]. However, studies which attempted to link these phenotypic and life history traits with molecular data concluded that the latter provide deeper insight into the evolutionary history of *Haemosporidia*, and allow the identification of cryptic species which cannot be distinguished using only microscopy observations [9,10]. The increasing availability of molecular data has enabled numerous studies of *Haemosporidia *phylogeny, improving our understanding of the evolutionary history of malaria parasites.

To date, five *Plasmodium *species have been shown to cause human malaria: *P*. *falciparum*, *P. vivax, P. malariae, P. ovale *and *P. knowlesi *[11-14]. *P. falciparum *has stimulated the interest of the scientific community, largely because it is the most virulent, but also because of the very high A+T contents of its genome. Indeed, it has an average content of 80% A+T over its nuclear genome, with intergenic regions frequently displaying more than 90% A+T [15]. Furthermore, the question of the origin of *P. falciparum *is intriguing: its 18 S rRNA gene diverges strikingly from that of the other *Plasmodium *species which infect primates, of which four cause human malaria [16-19]. Another parasite of interest is *P. reichenowi*, which infects chimpanzees. *P. reichenowi *has long been the only known close relative of *P*. *falciparum *[6,18-24]. However, since 2009, it has been recognized that both *P. falciparum *and *P. reichenowi *belong to a wider monophyletic lineage of parasites specialized in the infection of great ape hosts: gorilla, chimpanzee, bonobo and human [25-28].

**Figure 1:**
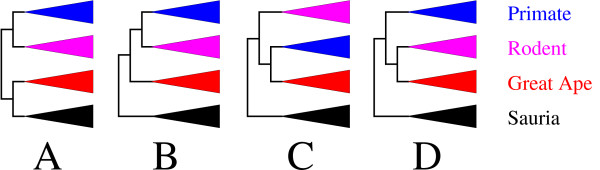
**Competing hypotheses for the origin of Plasmodium falciparum and great ape malaria parasites**. A: The avian origin hypothesis [16-19,21,29-35]. 1B: Great ape parasites sister-group to all other mammal parasites [6,20-22,24,25,27,28,36]. 1C: Close relationship with primate malaria parasites [14]. 1D: Close relationship with rodent malaria parasites [23,27].

Despite intense efforts to obtain accurate molecular phylogenies, the position of *P. falciparum *and of great ape malaria parasites within *Haemosporidia *remains unclear. In recent decades, many molecular phylogenies have been produced which clustered *P. falciparum *(alone, or with *P. reichenowi*) closer to avian than to mammalian parasites (Figure 1A). This led to the hypothesis of a recent switch from avian to human (and great ape) hosts, rather than a shared ancestry of *P. falciparum *and other mammalian malaria parasites [16-19,21,29-35]. However, it has recently been recognized that, in these early works, the clustering of *P. falciparum *with avian malaria parasites may have resulted mainly from stochastic noise due to the insufficient number of sequences analyzed [35]. More recent studies relied on larger data sets and demonstrated that all malaria parasites known to exclusively infect mammals, including *P. falciparum*, are monophyletic [6,20-28,36]. Hence, this monophyletic clade of mammalian malaria parasites includes (i) a lineage infecting great apes [25-28], (ii) a distantly related and less specialized lineage infecting primates [11], (iii) a third lineage of rodent parasites [6,37], and (iv) species of the *Hepatocystis *genus infecting bats and primates [5,6,21]. Most studies inferred great ape parasites to be a sister-group to all other mammal parasites [6,20-22,24,25,27,28,36] (Figure 1B). However, a few studies yielded contradictory results. Some suggested that great ape parasites could be closer to other primate parasites [14] (Figure 1C), while others suggested that great ape parasites could be related to rodent parasites [23,27] (Figure 1D).

In the present study, we distinguished between these mutually exclusive hypotheses to identify the origin of *Plasmodium falciparum *and other great ape parasites. We analyzed three genes from 33 publicly available complete *Haemosporidian *mitochondrial genomes, as well as 51 additional sets of one to three mitochondrial genes from *Hepatocystis *and *Plasmodium *species infecting various mammalian and saurian hosts. Concatenated nucleotide and amino-acid alignments were analyzed using various probabilistic models of sequence evolution, applying both maximum-likelihood (ML) and Bayesian inference (BI) methods. Statistical measurements of fit and posterior predictive experiments enabled the adequacy of model assumptions to the data to be checked. We also evaluated the robustness of the obtained phylogeny to the taxonomic sampling by removing and adding taxa. Our phylogenetic analyses provide robust support for a close relationship between great ape and rodent parasites (Figure 1D).

## Results and Discussion

### Preliminary measures

In this study, we analyzed coding genes from the 33 complete *Haemosporidian *mitochondrial genomes available in 2009 (see Methods and Additional file 1, Table S1). Each genome includes only three coding genes. In this section, we describe the general features of the resulting alignments.

In the three concatenated genes and 33 taxa data set, 54% of the 1099 amino-acid sites, and 51% of the 3308 nucleotide sites were constant. The nucleotide and amino-acid alignments are thus highly conserved. Genes displayed the typical pattern in which substitution rates are higher at the third and first codon positions, and lower at the second codon positions. The first and second codon positions are highly conserved (55% and 77% of constant positions, respectively) and the saturation plot indicates that they are slightly saturated (slopes of 0.19 and 0.89 respectively, Additional file 2, Figure S1). Although third codon positions evolved more rapidly and are thus more saturated (20% of constant sites, slope of 0.09, Additional file 2, Figure S1), another standard test for saturation (PAUP 4.0 [38], partition-homogeneity test by codon position) indicated that they are not significantly more saturated than the first and second codon positions (*p *= 1).

The nucleotide data set had a 74% A+T content and was compositionally homogeneous (*p *= 0.83, PAUP *χ*^2 ^test of compositional homogeneity across taxa). However, the amount of A+T and the compositional homogeneity differed strikingly depending on the codon position. First and second codon positions displayed relatively low A+T contents (68% and 64% respectively) and were homogeneous (*p *= 1, PAUP *χ*^2 ^test of compositional homogeneity across taxa). In contrast, third codon positions had a high A+T content (89%) and were compositionally heterogeneous (*p *= 0, PAUP *χ*^2 ^test of compositional homogeneity across taxa). This difference in composition between codon positions suggests that the typical drift of *Haemosporidian *species toward A+T richness was negatively selected at first and second codon positions and less constrained at third positions, most likely due to constraints at the protein level.

### Standard phylogenetic analyses

Phylogenetic reconstructions in this section were obtained with the most widely used models, which have both maximum-likelihood (ML) and Bayesian implementations. MrAIC [39] estimated that, among 56 models of nucleotide substitution, the best AIC score [40] was achieved by the most general homogeneous and reversible model (GTR, "general time reversible" [41]), combined with four discrete gamma categories of "rates across sites" [42], plus an additional rate category for invariant sites (model *GTR_nt _*+ Γ_4 _+ *I*, where subscript *nt *stands for nucleotides). This model was also selected by MrAIC with individual codon positions. We used the PhyloBayes 3.0 software [43] for Bayesian analyses, which does not implement the invariant rate category. Bayesian analyses of nucleotide alignments were thus performed under the *GTR_nt _*+ Γ_4 _model.

**Figure 2:**
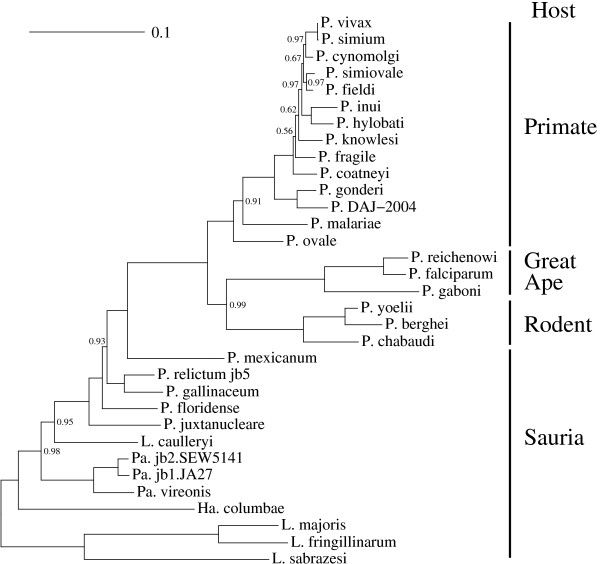
**Phylogeny of mitochondrial genes of 33 Haemosporidian species**. Bayesian phylogenetic reconstruction under the *GTR_nt _*+ Γ_4 _model, using PhyloBayes 3.0 [43]. *P. falciparum *and two of its relatives infecting great ape hosts, *P. reichenowi *and *P. gaboni*, form a monophyletic clade with three rodent parasites, *P. yoelii*, *P. berghei *and *P. chabaudi *(posterior probability *PP *= 0.99). Posterior probabilities equal to 1 were removed. Abbreviations "P.": *Plasmodium *species, "He.": *Hepatocystis *species, "Ha.": *Haemoproteus *species, "Pa.": *Parahaemoproteus *species, "L.": *Leucocytozoon *species.

ML and Bayesian analyses of the nucleotide data set under *GTR_nt _*+ Γ_4 _+ *I *and *GTR_nt _*+ Γ_4 _models, respectively, strongly supported a clade containing the 20 mammal infecting *Plasmodium *species (Figure 2) [6,20-25,27,28,36]. The mammal malaria parasite clade comprises three strongly supported main lineages. One lineage is specialized in infecting great ape hosts and includes *P. falciparum, P. reichenowi *and *P. gaboni *[25-28]. The second lineage is characterized by African and Asian primate hosts and comprises 14 *Plasmodium *species [11]. The third lineage includes *P. berghei*, *P. yoelii *and *P. chabaudi*, the African rodent malaria parasites [37]. Most interestingly, the two lineages of great ape and rodent parasites clustered together with strong posterior probability (*PP *= 0.99, Figure 2). ML analysis also yielded significant support for this clade (Shimodaira-Hasegawa-like support *SH *= 0.93, bootstrap support *BS *= 0.82, Table 1).

**Table 1:** Support dependency on the model assumptions

Model	Type of Data	Statistical Support		
		*PP*	*SH*	*BS*
*GTR_nt _*+ Γ_4 _(+*I*)				
*CAT *+*GTR_nt _*+ Γ_4_	Nucleotide	0.995	*	*
*GTR_nt _+BP + *Γ _4_		0.949	*	*
			
*JTT + *Γ_4 _(+*I*)		0.919	0.46	0.416
*GTR_aa _*+ Γ_4_		0.978	*	*
*CAT *+ Γ_4_	Amino Acid	0.629	*	*
*CAT +JTT *+ Γ_4_		0.802	*	*
*CAT +BP *+ Γ_4_		0.747	*	*

Support dependency of the great ape and rodent parasites monophyly on assumptions of various probabilistic models of substitution (*GTR_nt_*, *GTR_aa _*and *JTT*: single matrix model, *CAT*: site heterogeneous mixture model, *BP *: time heterogeneous model). Rates across site model components are defined as + Γ_4 _+ *I *under maximum-likelihood (ML) and as + Γ_4 _under Bayesian inference (BI) methods. *PP*: Posterior probability (BI), *SH*: Shimodaira-Hasegawa-like support (ML), and *BS*: bootstrap support (ML). "*": not applicable.

First and second codon positions, and third codon positions were analyzed separately, using the same models of nucleotide evolution as previously. Both data sets supported, although weakly, a clade containing great ape and rodent malaria parasites, hereafter denoted "monophyly of great ape and rodent parasites" (Additional file 3, Table S2, column "Rodent").

Finally, ProtTest [44] determined that the best fit to the amino-acid alignment was provided by the *JTT *+ Γ_4 _+ *I *+ *F *model [45]. We used the *JTT *+ Γ_4 _model for the Bayesian analysis of the amino-acid alignment. The monophyly of great ape and rodent parasites received weak SH and bootstrap support (*SH *= 0.46, *BS *= 0.41), but relatively high posterior probability (*PP *= 0.92, Table 1).

*SH *and *BS *supports are more conservative than posterior probabilities, which are generally expected to be higher [46,47]. Moreover, given the high level of conservation of protein and individual codon position alignments (see above), the complete nucleotide alignment is expected to provide more phylogenetic signal and higher supports. Thus, all previous results are congruent, with differing but explainable levels of confidence, and support a monophyly of great ape and rodent parasites.

### Assessment of model violation and robustness to the model choice

To evaluate the influence of potential model violations, we used improved Bayesian models implemented in PhyloBayes 3.0 software [43]. The fits of these additional models to the data were measured using cross-validated likelihood (see Methods and Additional file 4, Table S3). Moreover, we applied posterior predictive tests which measure the model ability to accurately reproduce observed features of the data (see Methods and Additional file 5, Table S4). In this section, we only comment on experiments where posterior predictive tests were not rejected (*i.e*. there was significant violation of model assumptions). In this case, we conclude that a specific feature of the data is correctly anticipated by the model assumptions.

We wanted to evaluate the potential effects of site saturation on our estimates. Site-heterogeneous mixture models such as "CAT" [48] (see Methods) efficiently deal with violations caused by high saturation levels [48,49]. However, this model might lack resolution power, especially in the case of small data sets [48]. In addition, we wanted to measure the potential effect of compositional biases on our estimates. Consequently, we analyzed data sets under the time-heterogeneous model "BP" [50,51], which is designed to deal with compositional heterogeneity across taxa (see Methods).

#### Saturation in the nucleotide alignment

Among the three additional models considered, *CAT *+ *GTR_nt _*+ Γ_4 _[43] (see Methods) yielded the best fit to the nucleotide data set, and it outperformed *GTR_nt _*+ Γ_4 _by 103 points of cross-validated likelihood (Additional file 4, Table S3). Moreover, posterior predictive tests showed that this model correctly anticipated the level of saturation of the nucleotide data set (*p *> 0.28, Additional file 5, Table S4). This suggests that the *CAT *+ *GTR_nt _*+ Γ_4 _model is not misled by site saturation. *CAT *+ *GTR_nt _*+ Γ_4 _strongly supported the monophyly of great ape and rodent parasites, considering either all codon positions (*PP *= 0.99, Table 1), first and second (see Additional file 6, Figure S2), or third codon positions (*PP *= 0.92 and *PP *= 0.65 respectively, Additional file 3, Table S2).

#### Compositional heterogeneity in the nucleotide alignment

Third codon positions were compositionally heterogeneous (see above *χ*^2 ^tests), and they carried 54% of variable sites. Hence, potential convergence of sequence compositions could have misled the previously used time-homogeneous models [50-52]. Interestingly, posterior predictive tests showed that the compositional heterogeneity across taxa of first and second codon positions was correctly anticipated under the time-homogeneous models *GTR_nt _*+ Γ_4 _and *CAT *+ *GTR_nt _*+ Γ_4 _(*p *> 0.44, Additional file 5, Table S4), suggesting that these models are relatively robust to compositional changes in this data set.

The time-heterogeneous model *GTR_nt _*+ *BP *+ Γ_4 _[50] (see Methods) explicitly accounts for variations in composition across taxa. It correctly anticipated the observed compositional heterogeneity, considering either all, first and second, and third codon positions (*p *> 0.11, Additional file 5, Table S4), suggesting that this model is unlikely to be misled by compositional heterogeneity across taxa. The monophyly of great ape and rodent parasites was strongly supported under the *GTR_nt _*+ *BP *+ Γ_4 _model, considering either all codon positions (*PP *= 0.95, Table 1), first and second, or third codon positions (*PP *= 0.99 and *PP *= 0.72 respectively, Additional file 3, Table S2).

#### Model violations in the amino-acid alignment

According to the analysis of the amino-acid data set, methodological bias may arise from the use of the universal replacement model *JTT*. The peculiar A+T rich composition of *Haemosporidian *genes could lead to slightly different estimations for the exchange rate parameters and hence alter the probability of clustering great ape and rodent parasites together. Accordingly, we used the *GTR_aa _*model (where subscript *aa *stands for amino acids) which does not rely on a pre-estimated replacement matrix like *JTT*. The *GTR_aa _*+ Γ_4 _model strongly supported the monophyly of great ape and rodent parasites (*PP *= 0.98, Table 1).

Cross-validations indicated that, among the 12 alternative models, the site-heterogeneous model *CAT *+ *JTT *+ Γ_4 _[43] (see Methods) provided the best fit to the amino-acid data set (Additional file 4, Table S3). According to posterior predictive tests, this model correctly anticipated the saturation level observed in the data (*p *> 0.07, Additional file 5, Table S4). Moreover, the site- and time- heterogeneous model *CAT *+ *BP *+ Γ_4 _[51] (see Methods) correctly anticipated the level of saturation of the amino-acid data set (*p *= 0.41). However, the posterior predictive test for compositional heterogeneity across taxa was rejected under *CAT *+ *BP *+ Γ_4 _(*p *= 0.02), although as expected, this model anticipated compositional heterogeneity better than do time-homogeneous models (*p *= 0.001, Additional file 5, Table S4). Both the two last models moderately supported the monophyly of rodent and great ape parasites (*PP *= 0.80 and *PP *= 0.75 under *CAT *+ *JTT *+ Γ_4 _and *CAT *+ *BP *+ Γ_4 _respectively, Table 1). In both cases, few variable amino-acid positions are interpreted under highly parameter rich models, and moderate support is therefore to be expected.

Most importantly, all results in this section are congruent with our initial estimate. This suggests that measured model violations do not significantly bias the relationship of great ape parasites among mammal parasites. In other words, the monophyly of great ape and rodent parasites appears to be robust to the choice of the model, as well as to its assumptions and dimensionality.

### Robustness to taxon sampling

To avoid possible biases resulting from *an ad *hoc set of sequences, it is important to assess the robustness of this phylogenetic association with respect to taxon selection, which was achieved by analyses of 30 different taxonomic samples. Among previously considered data sets, the complete nucleotide data set had the highest number of variable positions to interpret (1627), and our experiments showed that it is unlikely to induce strong model violation, whatever the phylogenetic model considered. Hence, in the following sections, we present phylogenetic analyses of complete nucleotide alignments performed under models *GTR_nt _*+ Γ_4 _+ *I *(ML) and *GTR_nt _*+ Γ_4 _(Bayesian inference).

#### Robustness to taxon removal

First, we checked the influence on phylogenetic reconstructions of the selected great ape parasites. According to the initial taxon selection, this lineage includes *P. falciparum*, *P. reichenowi *and *P. gaboni*. Six additional combinations of these three taxa were devised (see Methods). The weakest (but still relatively high) support for the monophyly of great ape and rodent parasites was obtained when *P. reichenowi *was considered as the only representative of its lineage (*PP *= 0.93, *SH *= 0.78, *BS *= 0.68, Additional file 7, Table S5). Second, we devised six combinations of the three rodent parasites, *P. berghei*, *P. yoelii *and *P. chabaudi*. The data set with *P. berghei *as the only representative of its lineage yielded the weakest support for the monophyly of great ape and rodent parasites (*PP *= 0.56, *BS *= 0.52, Additional file 7, Table S5), and the ML tree weakly supported the alternative hypothesis of a monophyly of primate and rodent parasites (*i.e*. Figure 1B, *SH *= 0.05). Third, six combinations of primate parasites were considered. Only the combination with African primate parasites (*P. gonderi *and *P. DAJ-2004*) as the only representatives of their lineage supported the alternative hypothesis of a monophyly of primate and rodent parasites (*i.e*. Figure 1B, *PP *= 0.85, *SH *= 0.26, *BS *= 0.48, Additional file 7, Table S5). The five other combinations of primate parasites supported the monophyly of great ape and rodent parasites (*PP *> 0.96, *SH *> 0.60, *BS *> 0.71, Additional file 7, Table S5). Finally, we investigated the robustness to the taxon composition of the outgroup (*i.e*. mammal and saurian parasites were considered as ingroup and outgroup, respectively). All six devised outgroups yielded high support for the monophyly of great ape and rodent parasites (*PP *> 0.98, *SH *> 0.64, *BS *> 0.74, Additional file 7, Table S5).

Hence, with the exception of three taxonomic samples, in which (i) *P. reichenowi*, (ii) *P. berghei *and (iii) *P. gonderi *and *P. DAJ-2004 *were considered as the only representatives of their respective lineages, all other 21 combinations of taxa provided good support for the association of great ape and rodent parasites (*PP *> 0.96, *SH *> 0.60, *BS *> 0.71, Additional file 7, Table S5).

#### Robustness to taxon addition

First, (i) 8 CytB genes from great ape parasites, (ii) 10 pairs of CytB and Cox1 genes from rodent parasites and, (iii) 27 pairs of CytB and Cox1 genes from *Plasmodium *species infecting a wide range of sauria hosts (Additional file 8, Table S6) were added in turn to the initial 33-taxon data set. The association of great ape and rodent parasites was still strongly supported (*PP *> 0.99, *SH *> 0.77, *BS *> 0.78, Additional file 7, Table S5), and the lineages of great ape, rodent, and mammal parasites were each still shown to be monophyletic (*PP *= 1).

Second, all previous taxa were analyzed together (33 taxa + 8 great ape + 10 rodent + 27 saurian parasites), yielding a 78-taxon tree. The monophyly of great ape and rodent parasites was still strongly supported (*PP *= 0.99, *SH *= 0.86, *BS *= 0.76, Additional file 7, Table S5).

Third, we added six *Hepatocystis *species to the initial 33-taxon nucleotide data set. These six parasites were monophyletic (*PP *= 1). They clustered within the clade of mammal parasites, which was then composed of four monophyletic main lineages. The monophyly of great ape and rodent parasites was weakly supported (*PP *= 0.40, *SH *= 0.05, *BS *= 0.38), but this low support was entirely due to high uncertainty with respect to the position of *Hepatocystis *within mammalian malaria parasites. Indeed, *Hepatocystis *were located, with weak support, in five positions on trees in which great ape and rodent parasites were located close together (*e.g. Hepatocystis *as a sister-group to great ape parasites: *PP *= 0.28, *BS *= 0.28, or to rodent parasites: *PP *= 0.31, *BS *= 0.29). We evaluated posterior and bootstrap support for the great ape being close to, but not necessarily monophyletic with, rodent parasites (*i.e*. the great ape plus rodent parasite lineage could also include *Hepatocystis*). When support was summed over the three possible positions of *Hepatocystis *relative to the association of great ape and rodent parasites, then parasites of great apes and of rodents were located close together with strong support (*PP *= 0.99 and *BS *= 0.87, Additional file 7, Table S5).

**Figure 3:**
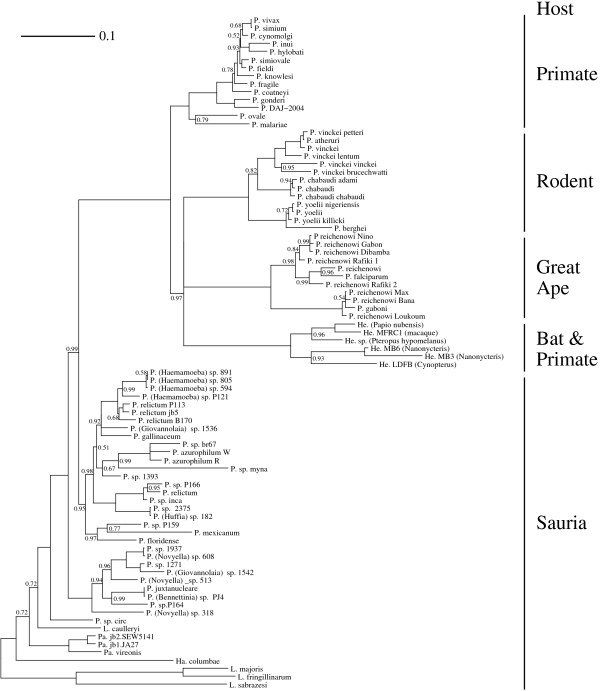
**Phylogeny of 84 Haemosporidian species**. Bayesian phylogenetic reconstruction under the *GTR_nt _*+ Γ_4 _model, using PhyloBayes 3.0 [43]. Posterior probabilities equal to 1 were removed, edges with posterior probability *PP *< 0.5 were collapsed. Abbreviations "P.": *Plasmodium *species, "He.": *Hepatocystis *species, "Ha.": *Haemoproteus *species, "Pa.": *Parahaemoproteus *species, "L.": *Leucocytozoon *species.

Fourth, the six *Hepatocystis *species were analyzed simultaneously with all the previous 78 taxa, yielding an 84-taxon tree (Figure 3). A strict monophyly of great ape and rodent parasites was weakly supported (*PP *= 0.28, *SH *= 0, *BS *= 0.31), due to high uncertainty with respect to the position of *Hepatocystis *(*e.g. Hepatocystis *as a sister-group to great ape parasites: *PP *= 0.36, *BS *= 0.25, or to rodent parasites: *PP *= 0.35, *BS *= 0.25). However, disregarding the exact position of *Hepatocystis*, great ape and rodent parasites were located close together with high support (*PP *= 0.99, *BS *= 0.75, Additional file 7, Table S5).

Thus, all six previous additions of mitochondrial genes did not alter the result indicating a likely close phylogenetic relationship of great ape and rodent parasites. Overall, statistical supports averaged over the 30 taxonomic samples considered in this section showed a robust relationship of great ape and rodent parasites . This suggests that this relationship does not depend solely on the selection of the taxa we considered here. However, the uncertainty concerning the exact position of *Hepatocystis *species within mammal parasites challenges the above mentioned monophyly of a clade only comprising parasites of great apes and of rodents, because it would be possible for *Hepatocystis *to cluster within that clade. Nonetheless, whatever the true position of *Hepatocystis *may be, it does not contradict our main result indicating that great ape parasites are unlikely to be a sister-group to all other mammal parasites [6,20-22,24,25,27,28,36] (Figure 1B), but instead, probably share a more recent common ancestor with rodent parasites [23,27] (Figure 1D).

### Comparison with previous studies

In previous studies, the hypothesis of great ape parasites being a sister-group to all other mammal parasites was defended by analyses of mitochondrial genes [6,21,22,24,27,35] or complete mitochondrial genomes [24,25,28], of nuclear coding genes [53] and ribosomal RNA [20], or by combining genes from nuclear and mitochondrial genomes with genes from the apicoplast genome [36]. Moreover, considering different rooting assumptions breaking the monophyly of mammal parasites, this result was also obtained through analyses of nuclear 18 S rRNA [11,18], nuclear genes [29,32,34] and mitochondrial *cytochrome b *genes [21].

Most of these studies of the *Haemosporidia *phylogeny relied on a single gene, and only a few taxa data sets, which might lack phylogenetic signal [35]. In contrast, two recent studies analyzed larger data sets. The first study analyzed a large number of taxa (40), but few concatenated genes (4) [36], whereas the second focused on a large number of genes (104), but considered very few taxa (8) [53]. Next, we suggest possible reasons for the disagreement between the results of these two studies and ours.

#### Comparison with a taxa-wide phylogenetic analysis

As a general guideline, wider taxon sampling usually helps to resolve phylogenies more accurately, provided enough genes are available to overcome stochastic noise, and are also sufficiently conserved to avoid systematic errors [54]. In line with this idea, Martinsen et al. [36] analyzed four concatenated genes for a relatively wide sample of 40 taxa. Among the previous works, their experimental conditions are thus the closest to ours. But, intriguingly, our results do not confirm theirs. We suggest that the disagreement between the two studies is due to several factors, the first being the differences in the phylogenetic markers analyzed.

**Figure 4:**
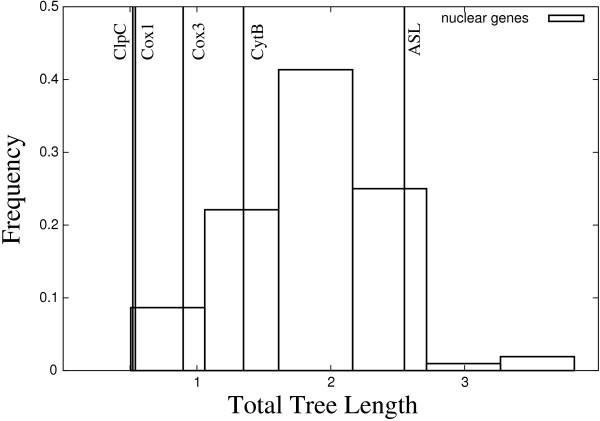
**Estimation of length of 8-taxa trees, for 109 genes**. Maximum-likelihood estimation of total lengths of 8- and 7- taxa gene trees, under *GTR_nt _*+ Γ_4 _+ *I *using PhyML 3.0 [47]. Taxa: *P. falciparum*, *P. reichenowi*, *P. vivax*, *P. knowlesi*, *P. berghei*, *P. chabaudi*, *P yoelii *and *P. gallinaceum*. Vertical lines: lengths of 8-taxa trees for CytB, Cox3 and Cox1 mitochondrial genes, and of 7-taxa trees, for ClpC and ASL (no sequence available for *P. reichenowi*, see accessions in Additional file 9, Table S7). Square boxes: distribution of lengths of 8-taxa trees over the 104 orthologous nuclear genes of Dávalos and Perkins [53].

Both Martinsen et al. [36] and our study considered CytB and Cox1 mitochondrial genes. However, Martinsen et al. [36] additionally analyzed *adenylosuccinate lyase *(ASL) and *caseinolytic protease C *(ClpC) genes, whereas we analyzed the third mitochondrial gene Cox3. In order to compare global rates of evolution between these genes, we measured the total lengths of gene trees [55], for a common sub set of eight taxa (*P. falciparum, P. reichenowi, P. vivax, P. knowlesi, P. berghei, P. chabaudi, P yoelii and P. gallinaceum*). Our values indicate that the ASL genes evolved 3 to 5 times faster than the slowest evolving genes: ClpC, Cox1 and Cox3 (Figure 4). The signal to noise ratio is expected to be higher for slowly evolving phylogenetic markers, and fast rates of evolution generally reduce the accuracy of inferred phylogenetic trees [49,56]. In addition, we considered ASL and ClpC genes of 18 and 27 taxa respectively, for taxonomic samples as close as possible to our originally selected 33 taxa (Additional file 9, Table S7). The rapidly evolving gene, ASL, did not support a monophyly of mammal parasites, suggesting strong systematic errors (Additional file 10, Figure S3). In contrast, the slow evolving gene, ClpC, supported this monophyly (*PP *= 0.99, *SH *= 0.94, *BS *= 0.51), but did not support any particular position of *P. falciparum *within mammal parasites (Additional file 11, Figure S4). However, a recent study of 14 ClpC genes supported a common ancestry of great ape and rodent parasites [57].

Moreover, most CytB, ClpC and ASL sequences analyzed by Martinsen et al. [36] were partial CDS. Thus, even considering four genes, their alignment covered 2334 nucleotide sites, representing 70% of the 3308 nucleotide sites considered in the present study. Finally, they considered fewer mammalian malaria parasites than we did (11 taxa in the study of Martinsen et al. [36], versus 20 to 44 taxa in our work), and they considered *P. falciparum *as the only representative of the lineage of great ape parasites, together with *P. vivax and P. knowlesi *as the only representatives of primate parasites. All these differences in experimental conditions (*i.e*. saturation of ASL gene, fewer sites, fewer mammal parasites close to *P. falciparum*) could together contribute to the difference between the results of Martinsen et al. [36] and ours.

#### Comparison with a genome-wide phylogenetic analysis

Dávalos and Perkins [53] extracted a set of 104 putative orthologous nuclear genes from the eight complete genomes of *Plasmodium *species sequenced to date (*P. falciparum, P. reichenowi, P. vivax, P. knowlesi, P. berghei, P. chabaudi, P yoelii and P. gallinaceum*). Their phylogenetic analyses of individual genes displayed discrepancies with respect to the inferred trees. Approximately half the 104 genes supported a monophyly of primate and rodent parasites (Figure 1B) [6,20-22,24,25,27,28,36]. Alternative hypotheses of a monophyly of great ape and primate parasites (Figure 1C) [14], and of great ape and rodent parasites (Figure 1D) [23,27], were each supported by nearly a quarter of these 104 genes. Interestingly, for the same reduced sample of eight taxa, our phylogenetic analyses of mitochondrial genes, taken separately, displayed comparable discrepancies (Table 2). Moreover, the supports obtained showed a near-random resolution of the internal branch of the 8-taxon individual gene trees. This suggests that, rather than showing a global preference of individual genes for the monophyly of primate and rodent parasites, the analysis of individual genes by Dávalos and Perkins [53] might have been strongly influenced by stochastic noise.

**Table 2:** Effects of reduced taxon and site sampling

		Supports for Great Ape parasites sister-group of:								
		
Number of Taxa	Genes	Rodent			Primate+Rodent			Primate		
		*PP*,	*SH*,	*BS*	*PP*,	*SH*,	*BS*	*PP*,	*SH*,	*BS*
	CytB	0.221,	*,	0.306	0.612,	0.29,	0.444	0.166,	*,	0.250
8 Taxa	Cox1	0.152,	*,	0.374	0.848,	0.45,	0.610	0.000,	*,	0.016
	Cox3	0.922,	0.63,	0.659	0.002,	*,	0.013	0.070,	*,	0.313
	Conc.	0.971,	0.58,	0.655	0.029,	*,	0.316	0.000,	*,	0.029
									
	CytB	0.911,	0.77,	0.548	0.074,	*,	0.225	0.013,	*,	0.009
33 Taxa	Cox1	0.022,	*,	0.336	0.976,	0.61,	0.606	0.001,	*,	0.043
	Cox3	0.985,	0.90,	0.801	0.003,	*,	0.011	0.012,	*,	0.092
	Conc.	0.999,	0.93,	0.821	0.001,	*,	0.174	0.000,	*,	0.004

Dependency of clade support on a reduced (8 Taxa) or extended (33 Taxa) taxon sampling, and on individual (CytB, Cox1 and Cox3 genes) or concatenated (Conc.) gene analyses. Models *GTR_nt _*+ Γ_4 _+ *I *and *GTR_nt _*+ Γ_4 _were applied to nucleotide alignments, under maximum-likelihood (ML) and Bayesian (BI) methods, respectively. Cells display support as [*PP*, *SH*, *BS*] with *PP*: posterior probability (BI), *SH*: Shimodaira-Hasegawa-like support (ML), and *BS*: bootstrap support (ML). "*": not applicable. Main lineages of mammalian parasites are defined according to their host preference: "Rodent", "Primate" and "Great Ape" (see Additional file 1, Table 1).

Increasing the amount of signal by concatenating genes helps to alleviate the effects of stochastic noise. The analysis of the three concatenated mitochondrial genes, for the eight taxa, supported the monophyly of great ape and rodent parasites (*PP *= 0.97, *SH *= 0.58, *BS *= 0.65; Table 2). In contrast, the concatenation of the 104 nuclear genes yielded strong support for the monophyly of primate and rodent parasites [53] (Figure 1B). We estimated the total tree lengths of each of these 104 genes, as well as of their concatenation. First, mitochondrial genes evolved as slowly as the  fraction of the 104 nuclear genes which displayed the slowest rate of evolution (Figure 4). Second, total tree length estimated for the 104 gene concatenation indicated a fast average rate of evolution, about two times faster than that of the three mitochondrial genes. These observations confirm the fact that mitochondrial genes are well conserved, and corroborate the conclusion of Dávalos and Perkins [53] indicating that most of their 104 genes are highly saturated and evolved relatively fast. Thus, the monophyly of primate and rodent parasites (Figure 1B), obtained by the latter authors from a large concatenation of 104 genes, most likely results from systematic errors that may be due to the high saturation level of most genes [53,58], but also presumably to the small sample of only eight taxa [54].

#### Comparison with two corroborated studies

In contrast, our results support a common origin of great ape and rodent parasites (Figure 1D). This corroborates results of a recent study published by Perkins [23] who, to the best of our knowledge, was the first to mention this hypothesis. This author sequenced seven new mitochondrial genomes and reconstructed the phylogeny of a sample of 24 taxa. A similar phylogeny was also obtained from 38 partial CytB sequences [27]. However, the two previous studies did not discuss the robustness of this result, but instead suggested it should be considered with caution [23,27], as most previous studies of mitochondrial genes supported a monophyly of primate and rodent parasites (Figure 1B) [6,21,22,24,24,25,27,28,35].

However, in our results, this hypothesis never obtained significant statistical support: at most, it reached *PP *= 0.35 and *BS *= 0.57 with the 33-taxon amino-acid alignment (Additional file 3, Table S2, column "Primate+Rodent"), and it obtained averaged supports of  and  over the 30 additional taxon samples considered (Additional file 7, Table S5, column "Primate+Rodent").

Interestingly, each time the monophyly of great ape and rodent parasites (Figure 1D) was not significantly supported, the only alternative hypothesis which could not be statistically rejected was the monophyly of primate and rodent parasites (*i.e*. support for "Primate+Rodent" in Additional files 3 and 7, Tables S2 and S5, where *PP *> 0.05). Moreover, nine additional samples of 19 taxa were drawn so that trees would display long branches, and we obtained five trees in which great ape parasites were shown to be a sister-group to primate and rodent parasites (Additional file 12, Table S8). This suggests that the monophyly of primate and rodent parasites (Figure 1B), rather than the monophyly of great ape and rodent parasites (Figure 1D), might result from the effect of long branch attraction. Thus, the slight tendency of mitochondrial genes to weakly support a monophyly of primate and rodent parasites, along with differences in taxon sampling and gene selection, could explain the disagreements between our results and most previous studies.

## Conclusions

With special focus on the still unclear phylogenetic position of great ape parasites, which include *Plasmodium falciparum *[25-28], in this study, we explored the phylogeny of *Haemosporidian *species by analyzing their mitochondrial genes. We showed that these genes have evolved relatively slowly and are mostly compositionally homogeneous, which characterizes them as potentially accurate phylogenetic markers. Corroborating many results obtained over the past few years, we obtained a monophyly of mammalian malaria parasites. Within that clade, we observed four main host-specialized lineages of parasites: *Plasmodium *species infecting (i) primate, (ii) rodent and (iii) great ape hosts, and (iv) *Hepatocystis *species infecting bats and primates. The inferred relationships within host-specialized lineages of *Plasmodium *parasites are congruent with the literature [11,26,37]. *Hepatocystis *species have received little attention to date. According to our results, these parasites may have diverged within mammalian malaria parasites, but their exact origin remains unclear. Our results support a common ancestry of great ape and rodent parasites (Figure 1D). We showed that this phylogenetic relationship is robust to various experimental conditions, demonstrating that it is unlikely to arise from an artefact of tree reconstruction.

Our study focused on mitochondrial genes. Nevertheless, it is still unclear if mitochondrial genomes match the *Haemosporidian *species tree or not [23]. This question could be answered by comparing the respective phylogenies of the three genomes hosted by *Haemosporidian *species (*i.e*. phylogenies of the mitochondrial, nuclear and apicoplast genomes). However, appropriate data sets for phylogenetic reconstruction of nuclear and apicoplast genomes are not yet available. A successful strategy for resolving a gene- and taxa- wide phylogeny would involve targeted sequencing of identified slowly evolving genes from the apicoplast and nuclear genomes. Careful phylogenetic analyses of such new and accurate phylogenetic markers will likely help to definitely resolve the phylogenetic origins of *Plasmodium falciparum *and other great ape parasites.

## Methods

### Mitochondrial gene and protein alignments

Complete mitochondrial genomes of 33 *Haemosporidian *parasites [22,23,25,59-66] were downloaded from the NCBI website (Additional file 1, Table S1). Available taxa included four *Leucocytozoon *species infecting birds [23,66]. These parasites are the closest relatives of other already identified *Haemosporidia *[6], and were used as outgroups. We collected mitochondrial genomes of four *Haemoproteus *and *Parahaemoproteus *species [23,62], and of five *Plasmodium *species [22,23,62,65] infecting saurian hosts (birds and reptiles). We collected a set of 20 mitochondrial genomes of mammal malaria parasites, including those of three rodent [59], three great ape [25,60,61] and 14 primate parasites [22,63,64].

Mitochondrial genomes of *Haemosporidian *species are vestigial and have a typical length of 6, 000 base pairs [67,68]. They form linear concatemers, each repeated unit encoding fragments of ribosomal genes together with three coding genes involved in the electron transport chain: *cytochrome b *and *cytochrome oxidase subunits I *and *III *(denoted as CytB, Cox1 and Cox3, respectively). Both nucleotide coding sequences and their translations into amino acids were retrieved according to annotated CDS. No annotation of the mitochondrial genes was available for *Plasmodium relictum jb5*, *Parahaemoproteus jb1.JA27 *and *Parahaemoproteus jb2.SEW5141*. In this case, gene sequences were extracted manually and unambiguously, given the high conservation level of start and end positions shared by all 30 other annotated genes.

Nucleotide sequences were aligned using MACSE (Ranwez V, Harispe S, Delsuc F, and Douzery EJP, "MACSE: Multiple Alignment of Coding Sequences accounting for frameshifts and stop codons", manuscript in preparation). This method computes the alignment of coding nucleotide sequences with respect to their possible translations. It attempts to minimize the occurrence of frameshifts and stop codons. We applied the relevant codon table, as indicated on the NCBI website [69]. MACSE identified and corrected three long frameshifts in the Cox3 gene of *P. berghei*, and in CytB genes of *Ha. columbae *and *L. caulleryi*. These frameshifts resulted in erroneous translations of the corresponding publically available amino-acid sequences. Consequently, we used the translated alignment computed by MACSE, rather than the alignment of the official NCBI translations. Individual gene and protein alignments were filtered with Gblocks 0.91 [70], and allowing a maximum of half gap states per site (option -*b*5 = *h*). Filtered alignments of nucleotide and amino-acid sequences were finally concatenated. This yielded two concatenations, one of 3308 nucleotide sites (number of nucleotide sites for CytB: 1125, Cox1: 1434, Cox3: 749), and one of 1099 amino-acid positions. The assembled data sets are available at the following URL: http://www.lirmm.fr/mab/blanquart

### Phylogenetic inferences

#### Description of phylogenetic models

Data sets were analyzed under various probabilistic models of molecular evolution. We applied the *JTT *[45] replacement model to the amino-acid data set. We applied the most general time reversible model *GTR_nt_*, where subscript *nt *denotes nucleotides, to the nucleotide data sets [41]. These substitution models were run using both maximum-likelihood (ML) and Bayesian inference (BI) methods. This allowed for the use of different statistical supports with different meanings, and comparison of the phylogenies estimated with the two approaches.

ML phylogenetic reconstructions were performed using PhyML 3.0 [47]. Irrespective of the substitution model (*GTR_nt_*, *JTT*) used for the analysis, the phylogenetic model additionally involved four discrete categories of gamma distributed rates across sites (denoted + Γ_4_, [42]), plus an invariant site category (denoted +*I*). The proportion of invariant sites and the shape parameter of the gamma distribution were estimated from the data. When the nucleotide data sets were analyzed, all eight free parameters of the *GTR_nt _*substitution models were estimated from the data (*GTR_nt _*+ Γ_4 _+ *I*, 10 degrees of freedom). In the case of the amino-acid alignment analyzed under *JTT*, stationary probabilities were set to empirical frequencies of amino acids measured over the whole data set (*JTT *+ Γ_4 _+ *I *+ *F*, 21 degrees of freedom). Note that these models were identified as the available ML models that best fit the sequence alignments, according to the AIC criterion [40].

Bayesian phylogenetic reconstructions were performed using PhyloBayes 3.0 [43]. For all Bayesian experiments performed in this study, two independent MCMC chains - each starting from a random point - were run for up to 100, 000 cycles. One MCMC sample was saved every 10 cycles, and the first 500 samples were discarded as "burnin". The eight free parameters of *GTR_nt _*and the amino-acid frequencies of *JTT *(19 free parameters), were estimated from the data.

We also applied more general and parameter rich models of evolution, implemented in a Bayesian framework. We used *GTR_aa_*, where subscript *aa *indicates *a GTR *model dedicated to amino-acid sequences. This model directly estimates the exchange rate parameters from the data (208 degrees of freedom). Models *JTT *and *GTR_aa _*homogeneously apply a single substitution model to the whole data set. However, in some cases, this parameterization is prone to violations by the data, resulting in wrong phylogenetic inferences [51]. Consequently, we applied the site-heterogeneous mixture model *CAT *to the amino-acid alignment, which implements a mixture of stationary probability vectors across sites [48]. The *CAT *model was combined with free (+*GTR_nt_*) or empirical (+*JTT*) relative exchange rates, applied to the nucleotide and amino-acid alignments, respectively. Both *CAT *+ *GTR_nt _*and *CAT *+ *JTT *models were combined with discretized gamma rates across sites (+ Γ_4_). Finally, we analyzed both nucleotide and amino-acid alignments under time-heterogeneous models of evolution. The *BP *model component allows for changes over time of the substitution model stationary probabilities and hence, estimates the compositional drift of the sequences [50]. We applied the *GTR_nt _*+ *BP *+ Γ_4 _[50] and the *CAT *+ *BP *+ Γ_4 _[51] models to the nucleotide and the amino-acid alignments, respectively.

Additional models with Bayesian implementations were compared using cross-validation (see below). In addition to the + Γ_4 _model of rate variation across sites, we applied a covarion model (+*COV*) which enabled us to estimate site specific rate variations (*i.e*. heterotachy) [71]. In addition to *JTT *and *GTR_aa_*, we considered the *MtREV *[72] empirical rate matrix. Finally, in addition to the mixture model *CAT*, we considered the empirical mixture models *UL*2 and *UL*3 [73]. These components allowed 13 and 3 models of evolution to be derived and applied to amino-acid and nucleotide alignments, respectively.

#### Cross-validation experiments

The fit of the models implemented in a Bayesian framework was estimated by cross-validation, as implemented in PhyloBayes 3.0 [43]. Ten replicate data sets were randomly drawn. The learning part of each replicate data set comprised 90% of the sites of the whole alignment. The 10% of remaining sites were used to compute the cross-validated likelihood. The tree topology was considered as a free parameter. Note that, for computational reasons, fits of time-heterogeneous models *GTR_nt _*+ *BP *+ Γ_4 _and *CAT *+ *BP *+ Γ_4 _were not evaluated.

#### Posterior predictive experiments

Model violations were measured by posterior predictive experiments, as implemented in PhyloBayes 3.0 [43]. We applied a test statistic measuring the compositional heterogeneity across taxa. The test statistic "composition" was defined as the maximum of the *χ*^2 ^distances separating each sequence composition from the composition of the whole data set [52]. We applied two test statistics to measure site saturation. The test statistic "site diversity" measures the mean state diversity across sites [48] (*e.g*. a constant site has a diversity of 1). The test statistic "homoplasy" considers the averaged number of convergence and reversion events per site, as displayed by inferred stochastic mapping [49]. A posterior predictive test compares the value *V_O _*of a test statistic measured given the observed data, to the distribution of that test statistic measured over simulated replicate data sets. Each replicate data set was simulated given an *a posteriori *drawn sample of parameters. The p-value indicates the probability of observing a test statistic as extreme as *V_O_*, under the null hypothesis stating that the model assumptions are true. Failure to reject a posterior predictive test indicates that the model assumptions allow to realistically reproduce the observation *V_O _*based on real data.

#### Saturation plot

Saturation of the phylogenetic signal of each codon position was illustrated by a saturation plot [74]. For each pair of taxa in an alignment, we plotted their "pairwise similarity distance" (*i.e*. y-coordinates: number of sites displaying different states, normalized by the alignment length), versus the distance separating these two taxa along the tree branches (*i.e*. x-coordinates: the sum of branch lengths from the two taxa to their common ancestor). We used a fixed tree topology estimated from all codon positions (Figure 2). Branch lengths and other model parameters were evaluated according to separate codon positions.

#### Estimation of tree node supports

Under ML analysis, statistical support of tree branches was estimated from 1000 bootstrap replicates and, in addition, using the Shimodaira-Hasegawa-like test (SH) implemented in PhyML 3.0 [47,75]. Bayesian analysis classically provides a collection of samples drawn from the *a posteriori *distribution. The posterior probability of observing a given phylogenetic association between two lineages is then approximated by its frequency among sampled trees. Given a monophyletic target lineage *A *(*e.g*. great ape parasites), we extracted from a tree collection the list of all its *N *different sister-groups *B_n _*(*e.g*. rodent parasites). We then computed the frequency : the posterior support of clade *A *+ *B_n _*(*e.g*. great ape plus rodent parasites). The same approach was used for the bootstrap support, but not for the SH support which applies only to clades that belong to the ML tree.

#### Taxon sampling

Let *A *and *B *each be a monophyletic lineage within a phylogenetic tree, and let them form a well-supported monophyletic clade *A *+ *B *according to an initial sample of taxa. To ensure that the target relationship between *A *and *B *does not result from stochastic noise (lack of signal) or systematic error (model violation), we checked its robustness to taxon sampling (*e.g*. [49]). Given a lineage *C *(possibly equal to *A *or *B*) including *k *taxa, we checked that every combination of 1 to *k *- 1 taxa of *C *yielded a congruent phylogeny with respect to the phylogenetic relationship *A *+ *B*. If lineage *C *was composed of too many taxa, we selected only a few relevant taxon combinations among all those available.

We focused on the robustness of the association of lineage *A*: great ape parasites, with lineage *B*: rodent parasites. Each of these two lineages was considered in turn as a sampled lineage *C*. According to our initial selection of 33 taxa, both these lineages were composed of 3 taxa and, 6 combinations of single or pairs of representatives were considered. We also considered in turn the group of primate parasites (14 taxa) and the saurian parasite outgroup (13 taxa) as sampled lineages *C*. For each of these sampled lineages, only 6 combinations of single or pairs of sub-groups were considered. For the 14 primate parasites, the 3 sub-groups were: (a) *P. malariae *and *P. ovale *(infecting humans), (b) *P. gonderi *and *P. DAJ-2004 *(African primate parasites), and (c) 10 *Plasmodium *species infecting Asian primates (Additional file 1, Table S1). For the 13 saurian parasites, the 3 sub-groups were (a) the 5 *Plasmodium *species, (b) the 4 *Haemoproteus *and *Parahaemoproteus *species, and (c) the 4 *Leucocytozoon *species (Additional file 1, Table S1). All these 24 sub data sets were obtained from the nucleotide alignment by simply discarding the relevant sequences without renewed aligning.

Finally, up to 51 malaria parasites were added to the initial selection of 33 taxa. We aligned all 84 taxa following the alignment procedure described above. Note that for CytB, 38 of the 51 additional sequences were partial CDS (see Additional file 8, Table S6). In order to retain more sites, complete and partial CytB genes were filtered separately with Gblocks, and then manually reassembled into an 84-gene alignment. All 6 additions of taxa to the 33-taxon data set were obtained from the whole concatenation of genes of the 84 taxa by discarding the relevant sequences.

## Authors' contributions

SB designed and conducted all the experiments. OG provided guidance throughout the study. Both authors contributed to the writing of the paper.

## Supplementary Material

Additional file 1 **Supplementary Table S1, Accession numbers of 33 mitochondrial genomes, species and host names**. Accession numbers of 33 complete mitochondrial genomes of *Haemosporidian *parasites, parasite names, and host names retrieved from NCBI annotations (*^b^*: host names complemented from Leclerc et al. 2004 [11]). "P.": *Plasmodium *species, "Ha.": *Haemoproteus *species, "Pa.": *Parahaemoproteus *species, "L.": *Leucocytozoon *species. Click here for file

Additional file 2 **Supplementary Figure S1, Saturation plot of codon positions**. Saturation plot of codon positions of the 33 taxa and 3 concatenated genes data set, computed with a Bio++ script [80]. Each dot represents the comparison of the similarity distance (y coordinate) versus the tree distance (x coordinate), for a pair of taxa. Tree branch lengths were estimated under the *GTR_nt _*+ Γ_4 _model (PhyloBayes 3.0, [43]), for 3 data sets corresponding to each codon position, and using the tree topology estimated from the whole nucleotide data set (Figure 2). Click here for file

Additional file 3 **Supplementary Table S2, Analyses of the 33 taxa and 3 mitochondrial gene data sets**. Dependency of clade support on codon positions ("Cod. pos."), amino-acid translation and on assumptions of various probabilistic models of substitution (*GTR_nt_*, *GTR_aa _*and *JTT*: single matrix model, *CAT*: site heterogeneous mixture model, *BP*: time heterogeneous model). Rates across site model components are defined as + Γ_4 _+ *I *under maximum-likelihood (ML) and as + Γ_4 _under Bayesian (BI) methods. Cells display support as [*PP*, *SH*, *BS*], with *PP*: posterior probability (BI), *SH*: Shimodaira-Hasegawa-like support (ML), and *BS*: bootstrap support (ML). "*": not applicable. Main lineages of mammal parasites are defined according to their host preference: "Rodent", "Primate" and "Great Ape" (see Additional file 1, Table S1). Click here for file

Additional file 4 **Supplementary Table S3, Fit of Bayesian models**. Cross-validation estimations of the fit of Bayesian models to the 33 taxa and the three concatenated gene data sets. Models applied to the nucleotide and amino-acid data set are compared to the best fitting ML models, *GTR_nt _*+ Γ_4 _and *JTT *+ Γ_4_, respectively. Models are defined according to their components. Substitution model: *GTR_nt_*, *GTR_aa_*, *MtREV *and *JTT*, exchange rate parameters; *CAT*, *UL*2 and *UL*3, site heterogeneous mixture models. Rates across sites models: + Γ_4_, discretized gamma rates (Yang 1994, [42]); +*COV*, covarion model (Tuffley and Steel 1998, [71]). Click here for file

Additional file 5 **Supplementary Table S4, p-values of posterior predictive tests performed on the 33 taxa and 3 mitochondrial gene data sets**. Data sets were analyzed under various probabilistic models of substitution (*GTR_nt_*, *GTR_aa _*and *JTT*: single matrix model, *CAT: *site heterogeneous mixture model, *BP*: time heterogeneous model, + Γ_4_: Rates across site model component). Posterior predictive test "Composition" measures compositional heterogeneity across taxa, "Site Diversity" and "Homoplasy" measure the level of saturation of the phylogenetic signal. "Cod. pos.": codon positions. "*": not applicable. Click here for file

Additional file 6 **Supplementary Figure S2, Phylogenetic tree of first and second codon positions analyzed under *CAT *+ *GTR *+ Γ_4_**. Bayesian phylogenetic reconstruction using PhyloBayes 3.0 [43]. The *CAT *+ *GTR_nt _*+ Γ_4 _substitution model was applied to first and second codon positions of the 33 taxa data set. *P. falciparum *and 2 of its relatives infecting great ape hosts, *P. reichenowi *and *P. gaboni*, formed a monophyletic clade with 3 rodent parasites, *P. yoelii*, *P. berghei *and *P. chabaudi *(posterior probability *PP *= 0.92). Posterior probabilities equal to 1 were removed. Click here for file

Additional file 7 **Supplementary Table S5, Robustness of the support to the removal and addition of taxa**. All codon positions were analyzed under *GTR *+ Γ_4 _and *GTR *+ Γ_4 _+ *I *models, for Bayesian and ML methods, respectively. Addition or removal of taxa to the complete nucleotide data set comprising 33 taxa and 3 concatenated genes, 3308 sites. Phylogenetic analyses were performed under models *GTR_nt _*+ Γ_4 _+ *I *and *GTR_nt _*+ Γ_4_, for maximum-likelihood (ML) and Bayesian (BI) methods, respectively. Cells display support as [*PP, SH, BS*], with *PP*: posterior probability (BI), *SH*: Shimodaira-Hasegawa-like support (ML), and *BS*: bootstrap support (ML). "*": not applicable. (*^a^*): *PP *and *BS *are summed over various positions of *Hepatocystis *species. Main lineages of mammal parasites are defined according to their host preference: "Rodent", "Primate" and "Great Ape" (see Additional file 1 and 8, Tables S1 and S6). "-" removal of species. "+" addition of species. "*P. fal*.": *P. falciparum*; "*P. rei*.": *P*. *reichenowi*; "*P. gab*.": *P. gaboni*; "*P. yoe*.": *P. yoelii*; "*P. ber*.": *P. berghei*; "*P. cha*.": *P. chabaudi*; "Hum": human primate parasites *P. malariae *and *P. ovale*; "Afr.": African primate parasites *P. gonderi *and *P. DAJ-2004*; "Asi": 10 Asian primate parasites; "Pla.": *Plasmodium *species infecting saurian hosts; "Hae.": *Haemoproteus *and *Parahaemoproteus *species; "Leu.": *Leucocytozoon *species; "Haemo." *Haemosporidian *species. Click here for file

Additional file 8 **Supplementary Table S6, Additional mitochondrial genes**. Accession numbers of 51, 41 and 1 additional CytB, Cox1 and Cox3 genes, respectively, and parasite and host names (^1 ^partial CytB genes). References: (*^a^*) Perkins and Schall (2002) [6]; (*^b^*) Perkins et al. (2007) [37]; (*^c^*) Cheesman et al. (2009) [76]; (*^d^*) Hall et al. (2005) [77]; (*^e^*) Escalante et al. (1998) [21]; (*^f^*) Seethamchai et al. (2008) [78]; (*^g^*) Martinsen et al. (2008) [36]; (*^h^*) Martinsen et al. (2007) [79]; (*^i^*) CytB + Cox1 + Cox3, Perkins (2008) [23]; (*^j^*) Rich et al. (2009) [26]. Abbreviation: "P.": *Plasmodium *species, "He.": *Hepatocystis *species. Click here for file

Additional file 9 **Supplementary Table S7, Accession numbers of ClpC and ASL genes**. Accession numbers of 27 ClpC and 18 ASL genes. (*^a^*): Taxa used for the estimation of the length of the 7-taxa tree. Click here for file

Additional file 10 **Supplementary Figure S3, Phylogenetic tree of 18 ASL genes**. Bayesian reconstruction under the *GTR_nt _*+ Γ_4 _model. Edges with *PP *< 0.9 were collapsed, and *PP *= 1 are not shown. The ASL phylogeny is not congruent with a monophyly of mammal malaria parasites. Click here for file

Additional file 11 **Supplementary Figure S4, Phylogenetic tree of 27 ClpC genes**. Bayesian reconstruction under the *GTR_nt _*+ Γ_4 _model. Edges with *PP *< 0.9 were collapsed, and *PP *= 1 are not shown. The ClpC phylogeny supports the monophyly of mammalian malaria parasites (*PP *= 0.99). Click here for file

Additional file 12 **Supplementary Table S8, Robustness of clade support in trees designed to display long branches**. Each of the 9 taxon samples comprised the three most distantly related *Leucocytozoon *species, the 14 primate parasites, and two single representatives of parasites of great apes and of rodents, respectively. In each case, 14 taxa were removed from the complete nucleotide data-set comprising 33 taxa and 3 concatenated genes, 3308 sites. Phylogenetic analyses were performed under models *GTR_nt _*+ Γ_4 _+ *I *and *GTR_nt _*+ Γ_4_, for maximum likelihood (ML) and Bayesian (BI) methods, respectively. Cells display support as follows: [*PP, SH, BS*], with *PP*: posterior probability (BI), *SH*: Shimodaira-Hasegawa-like support ("*": not applicable, ML), and *BS: *bootstrap support (ML). Main lineages of mammal parasites are defined according to their host preference: "Rodent", "Primate" and "Great Ape" (see Additional file 1, Table S1). "*P. fal*.": *P*. *falciparum*; "*P. rei*.": *P. reichenowi*; "*P. gab*.": *P. gaboni*; "*P. yoe*.": *P. yoelii; *"*P*. *ber*.": *P*. *berghei*; "*P. cha*.": *P. chabaudi*. Click here for file
