# Supplementary material for: Mitochondrial genes support a common origin of rodent malaria parasites and Plasmodium falciparum's relatives infecting great apes
Source: BMC Evol Biol. 2011 Mar 15;11:70. doi: 10.1186/1471-2148-11-70 (PMC3070646; doi:10.1186/1471-2148-11-70)
Supplement: Additional file 1 — Supplementary Table S1, Accession numbers of 33 mitochondrial genomes, species and host names. Accession numbers of 33 complete mitochondrial genomes of Haemosporidian parasites, parasite names, and host names retrieved from NCBI annotations (b: host names complemented from Leclerc et al. 2004 [11]). "P.": Plasmodium species, "Ha.": Haemoproteus species, "Pa.": Parahaemoproteus species, "L.": Leucocytozoon species. [file 1471-2148-11-70-S1.PDF]

Parasite Names	Accession	Known Hosts<br>Host names		Host Location
P. falciparum	AY282930	*Homo sapiens*	Great Ape	
P. gaboni	FJ895307	*Pan troglodytes*		
P. reichenowi	AJ251941	*Pan troglodytes*		
P. coatneyi	AB354575	*Macaca fascicularis*[b]	Other Primate	Asia
P. cynomolgi	AB434919	*Macaca sp.*[b]		
P. fieldi	AB354574	*Macaca sp.*[b]		
P. fragile	AY722799	*Macaca sp.*[b]		
P. hylobati	AB354573	*Hylobates moloch*[b]		
P. inui	AB354572	*Macaca cyclopis*		
P. knowlesi	AY722797	*Macaca fascicularis*		
P. simiovale	AY800109	*Macaca sinicia*		
P. simium	AY722798	*Saimiri sciureus*		
P. vivax	NC_007243	*Homo sapiens*		
P. DAJ-2004	AY800112	*Mandrillus sp.*		Africa
P. gonderi	AY800111	*Cercocebus sp.*[b]		
P. malariae	AB354570	*Homo sapiens*		
P. ovale	AB354571	*Homo sapiens*		
P. berghei	AF014115	*Grammomys surdaster*	Rodent	Africa
P. chabaudi	AF014116	*Thamnomys rutilans*		
P. yoelii	M29000	*Thamnomys rutilans*		
P. floridense	NC_009961	*Anolis sagrei*	Lizard	
P. mexicanum	AB375765	*Sceloporus mexicanum*		
P. gallinaceum	AB250690	*Gallus gallus*	Bird	
P. juxtanucleare	AB250415	*Crossoptilon crossoptilon*		
P. relictum jb5	AY733090	*Hemignathus virens*		
Ha. columbae	FJ168562	*Columba livia*		
Pa. jb1.JA27	AY733086	*Meliphaga lewinii*		
Pa. jb2.SEW5141	AY733087	*Lichenostomus frenatus*		
Pa. vireonis	FJ168561	*Vireo gilvus*		
L. caulleryi	AB302215	*Gallus gallus*		
L. fringillinarum	FJ168564	*Pipilo chlorurus*		
L. majoris	FJ168563	*Zonotrichia leucophrys*		
L. sabrazesi	AB299369	*Gallus gallus*		

Supplementary Table S1: **Accession numbers of 33 mitochondrial genomes, species and host names.** Accession numbers of 33 complete mitochondrial genomes of *Haemosporidian* parasites, parasite names, and host names retrieved from NCBI annotations ([b]: host names complemented from Leclerc et al. 2004 [11]). "P.": *Plasmodium* species, "Ha.": *Haemoproteus* species, "Pa.": *Parahaemoproteus* species, "L.": *Leucocytozoon* species.
