# Supplementary material for: Mitochondrial genes support a common origin of rodent malaria parasites and Plasmodium falciparum's relatives infecting great apes
Source: BMC Evol Biol. 2011 Mar 15;11:70. doi: 10.1186/1471-2148-11-70 (PMC3070646; doi:10.1186/1471-2148-11-70)
Supplement: Additional file 2 — Supplementary Figure S1, Saturation plot of codon positions. Saturation plot of codon positions of the 33 taxa and 3 concatenated genes data set, computed with a Bio++ script [80]. Each dot represents the comparison of the similarity distance (y coordinate) versus the tree distance (x coordinate), for a pair of taxa. Tree branch lengths were estimated under the GTRnt + Γ4 model (PhyloBayes 3.0, [43]), for 3 data sets corresponding to each codon position, and using the tree topology estimated from the whole nucleotide data set (Figure 2). [file 1471-2148-11-70-S2.PDF]

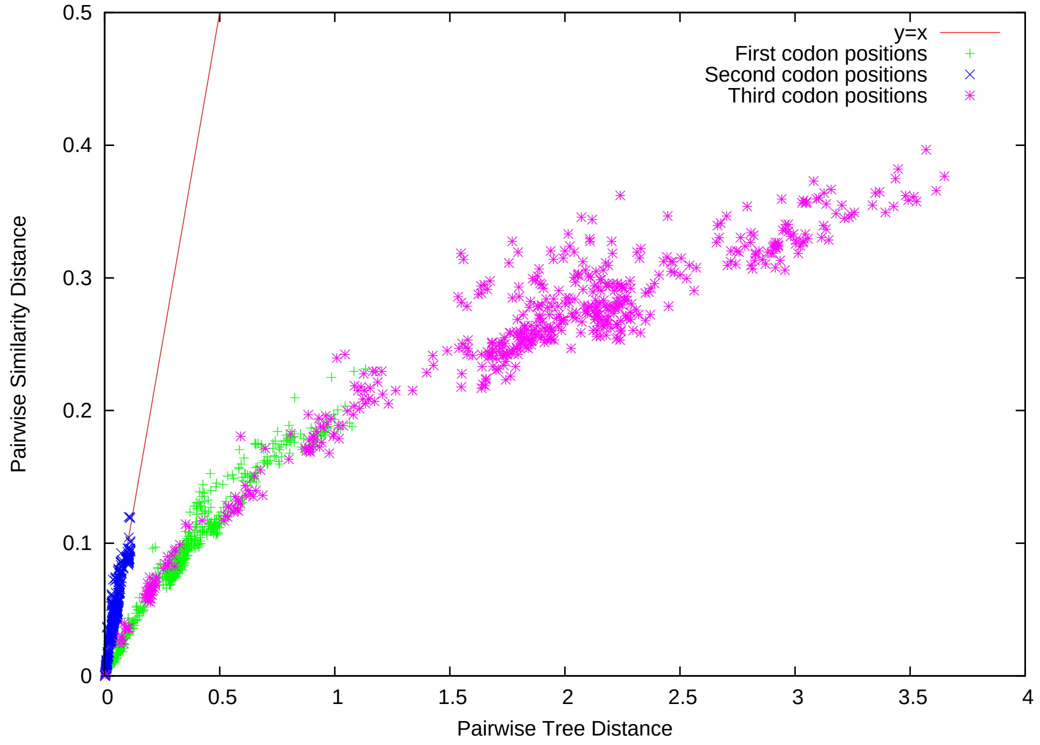

Supplementary Figure S1: **Saturation plot of codon positions.** Saturation plot of codon positions for the 33 taxa and 3 concatenated genes data set, computed with a Bio++ script [80]. Each dot represents the comparison of the similarity distance (y coordinate) versus the tree distance (x coordinate), for a pair of taxa. Tree branch lengths were estimated under the $GTR_{nt} + \Gamma_4$ model (PhyloBayes 3.0, [43]), for 3 data sets corresponding to each codon position, and using the tree topology estimated from the whole nucleotide data set (Figure 2).
