# Supplementary material for: Mitochondrial genes support a common origin of rodent malaria parasites and Plasmodium falciparum's relatives infecting great apes
Source: BMC Evol Biol. 2011 Mar 15;11:70. doi: 10.1186/1471-2148-11-70 (PMC3070646; doi:10.1186/1471-2148-11-70)
Supplement: Additional file 3 — Supplementary Table S2, Analyses of the 33 taxa and 3 mitochondrial gene data sets. Dependency of clade support on codon positions ("Cod. pos."), amino-acid translation and on assumptions of various probabilistic models of substitution (GTRnt, GTRaa and JTT: single matrix model, CAT: site heterogeneous mixture model, BP: time heterogeneous model). Rates across site model components are defined as + Γ4 + I under maximum-likelihood (ML) and as + Γ4 under Bayesian (BI) methods. Cells display support as [PP, SH, BS], with PP: posterior probability (BI), SH: Shimodaira-Hasegawa-like support (ML), and BS: bootstrap support (ML). "*": not applicable. Main lineages of mammal parasites are defined according to their host preference: "Rodent", "Primate" and "Great Ape" (see Additional file 1, Table S1). [file 1471-2148-11-70-S3.PDF]

Model	Type of Data Set	Supports for Great Ape parasites sister group of: Rodent	Primate+ Rodent	Primate
		PP, SH, BS	PP, SH, BS	PP, SH, BS
	Cod. pos. 1, 2, 3	0.999, 0.93, 0.821	0.001, *, 0.174	0.000, *, 0.004
GTR_{nt} $+\Gamma_4$ $(+I)$	Cod. pos. 1, 2	0.961, 0.78, 0.681	0.039, *, 0.316	0.000, *, 0.003
	Cod. pos. 3	0.793, 0.39, 0.587	0.146, *, 0.249	0.060, *, 0.104
	Cod. pos. 1, 2, 3	0.995,	0.003,	0.001,
CAT $+GTR_{nt}$ $+\Gamma_4$	Cod. pos. 1, 2	0.921,	0.057,	0.022,
	Cod. pos. 3	0.648,	0.108,	0.054,
	Cod. pos. 1, 2, 3	0.949,	0.005,	0.001,
GTR_{nt} $+BP$ $+\Gamma_4$	Cod. pos. 1, 2	0.987,	0.012,	0.000,
	Cod. pos. 3	0.717,	0.221,	0.062,
JTT $+\Gamma_4$ $(+I)$		0.919, 0.46, 0.416	0.081, *, 0.571	0.000, *, 0.007
GTR_{aa} $+\Gamma_4$		0.978,	0.022,	0.000,
CAT $+\Gamma_4$	Amino Acid	0.629,	0.352,	0.018,
CAT $+JTT$ $+\Gamma_4$		0.802,	0.195,	0.003,
CAT $+BP$ $+\Gamma_4$		0.747,	0.241,	0.011,

Supplementary Table S2: **Analyses of the 33 taxa and 3 mitochondrial gene data sets.** Dependency of clade support on codon positions ("Cod. pos."), amino-acid translation and on assumptions of various probabilistic models of substitution (GTR_{nt}, GTR_{aa} and JTT: single matrix model, CAT: site heterogeneous mixture model, BP: time heterogeneous model). Rates across site model components are defined as $+\Gamma_4 + I$ under maximum likelihood (ML) and as $+\Gamma_4$ under Bayesian (BI) methods. Cells display support as [PP, SH, BS], with PP: posterior probability (BI), SH: Shimodaira-Hasegawa-like support (ML), and BS: bootstrap support (ML). "*": not applicable. Main lineages of mammal parasites are defined according to their host preference: "Rodent", "Primate" and "Great Ape" (see Additional file 1, Table S1).
