# Supplementary material for: Mitochondrial genes support a common origin of rodent malaria parasites and Plasmodium falciparum's relatives infecting great apes
Source: BMC Evol Biol. 2011 Mar 15;11:70. doi: 10.1186/1471-2148-11-70 (PMC3070646; doi:10.1186/1471-2148-11-70)
Supplement: Additional file 4 — Supplementary Table S3, Fit of Bayesian models. Cross-validation estimations of the fit of Bayesian models to the 33 taxa and the three concatenated gene data sets. Models applied to the nucleotide and amino-acid data set are compared to the best fitting ML models, GTRnt + Γ4 and JTT + Γ4, respectively. Models are defined according to their components. Substitution model: GTRnt, GTRaa, MtREV and JTT, exchange rate parameters; CAT, UL2 and UL3, site heterogeneous mixture models. Rates across sites models: + Γ4, discretized gamma rates (Yang 1994, [42]); +COV, covarion model (Tuffley and Steel 1998, [71]). [file 1471-2148-11-70-S4.PDF]

Type of Data Set	Model	Score	SD
Nucleotide	$CAT + GTR + \Gamma_4$	+103.01	±9.15
	$CAT + GTR + COV + \Gamma_4$	+79.76	±9.44
	$GTR + COV + \Gamma_4$	−7.22	±6.46
Amino Acid	$CAT + JTT + \Gamma_4$	+17.88	±5.87
	$CAT + MTREV + \Gamma_4$	+16.49	±6.92
	$CAT + GTR + \Gamma_4$	+13.68	±9.25
	$CAT + GTR + COV + \Gamma_4$	+1.55	±12.97
	$GTR + \Gamma_4$	+1.24	±5.14
	$CAT + JTT + COV + \Gamma_4$	+1.07	±9.41
	$CAT + \Gamma_4$	−1.09	±6.27
	$MTREV + \Gamma_4$	−1.38	±4.11
	$CAT + MTREV + COV + \Gamma_4$	−3.25	±7.20
	$UL3 + \Gamma_4$	−20.10	±10.45
	$UL3 + COV + \Gamma_4$	−22.42	±8.20
	$UL2 + \Gamma_4$	−26.71	±8.77
	$UL2 + COV + \Gamma_4$	−27.66	±7.40

Supplementary Table S3: **Fit of Bayesian models.** Cross-validation estimations of the fit of Bayesian models to the 33 taxa and the three concatenated gene data sets. Models applied to the nucleotide and amino-acid data-set are compared to the best fitting ML models, $GTR_{nt} + \Gamma_4$ and $JTT + \Gamma_4$, respectively. Models are defined according to their components. Substitution model: GTR_{nt}, GTR_{aa}, $MtREV$ and JTT, exchange rate parameters; CAT, $UL2$ and $UL3$, site heterogeneous mixture models. Rates across sites models: $+\Gamma_4$, discretized gamma rates (Yang 1994, [42]); $+COV$, covarion model (Tuffley and Steel 1998, [71]).
