# Supplementary material for: Mitochondrial genes support a common origin of rodent malaria parasites and Plasmodium falciparum's relatives infecting great apes
Source: BMC Evol Biol. 2011 Mar 15;11:70. doi: 10.1186/1471-2148-11-70 (PMC3070646; doi:10.1186/1471-2148-11-70)
Supplement: Additional file 5 — Supplementary Table S4, p-values of posterior predictive tests performed on the 33 taxa and 3 mitochondrial gene data sets. Data sets were analyzed under various probabilistic models of substitution (GTRnt, GTRaa and JTT: single matrix model, CAT: site heterogeneous mixture model, BP: time heterogeneous model, + Γ4: Rates across site model component). Posterior predictive test "Composition" measures compositional heterogeneity across taxa, "Site Diversity" and "Homoplasy" measure the level of saturation of the phylogenetic signal. "Cod. pos.": codon positions. "*": not applicable. [file 1471-2148-11-70-S5.PDF]

Type of Data Set	Model	Posterior Predictive Test		
		Composition	Site Diversity	Homoplasy
Cod. pos. 1, 2, 3	$GTR_{nt} + \Gamma_4$	0.001	0.000	0.158
	$CAT + GTR_{nt} + \Gamma_4$	0.000	0.630	0.276
	$GTR_{nt} + BP + \Gamma_4$	0.122	0.000	*
Cod. pos. 1, 2	$GTR_{nt} + \Gamma_4$	0.737	0.000	0.036
	$CAT + GTR_{nt} + \Gamma_4$	0.447	0.643	0.111
	$GTR_{nt} + BP + \Gamma_4$	0.115	0.000	*
Cod. pos. 3	$GTR_{nt} + \Gamma_4$	0.004	0.000	0.372
	$CAT + GTR_{nt} + \Gamma_4$	0.000	0.421	0.302
	$GTR_{nt} + BP + \Gamma_4$	0.277	0.000	*
Amino Acid	$JTT + \Gamma_4$	0.001	0.000	0.026
	$GTR_{aa} + \Gamma_4$	0.001	0.000	0.031
	$CAT + JTT + \Gamma_4$	0.001	0.199	0.068
	$CAT + \Gamma_4$	0.001	0.390	0.541
	$CAT + BP + \Gamma_4$	0.017	0.416	*

Supplementary Table S4: **p-values of posterior predictive tests performed on the 33 taxa and 3 mitochondrial gene data sets.** Data sets were analyzed under various probabilistic models of substitution (GTR_{nt}, GTR_{aa} and JTT: single matrix model, CAT: site heterogeneous mixture model, BP: time heterogeneous model, $+\Gamma_4$: Rates across site model component). Posterior predictive test "Composition" measures compositional heterogeneity across taxa, "Site Diversity" and "Homoplasy" measure the level of saturation of the phylogenetic signal. "Cod. pos.": codon positions. "*": not applicable.
