# Supplementary material for: Mitochondrial genes support a common origin of rodent malaria parasites and Plasmodium falciparum's relatives infecting great apes
Source: BMC Evol Biol. 2011 Mar 15;11:70. doi: 10.1186/1471-2148-11-70 (PMC3070646; doi:10.1186/1471-2148-11-70)
Supplement: Additional file 6 — Supplementary Figure S2, Phylogenetic tree of first and second codon positions analyzed under CAT + GTR + Γ4. Bayesian phylogenetic reconstruction using PhyloBayes 3.0 [43]. The CAT + GTRnt + Γ4 substitution model was applied to first and second codon positions of the 33 taxa data set. P. falciparum and 2 of its relatives infecting great ape hosts, P. reichenowi and P. gaboni, formed a monophyletic clade with 3 rodent parasites, P. yoelii, P. berghei and P. chabaudi (posterior probability PP = 0.92). Posterior probabilities equal to 1 were removed. [file 1471-2148-11-70-S6.PDF]

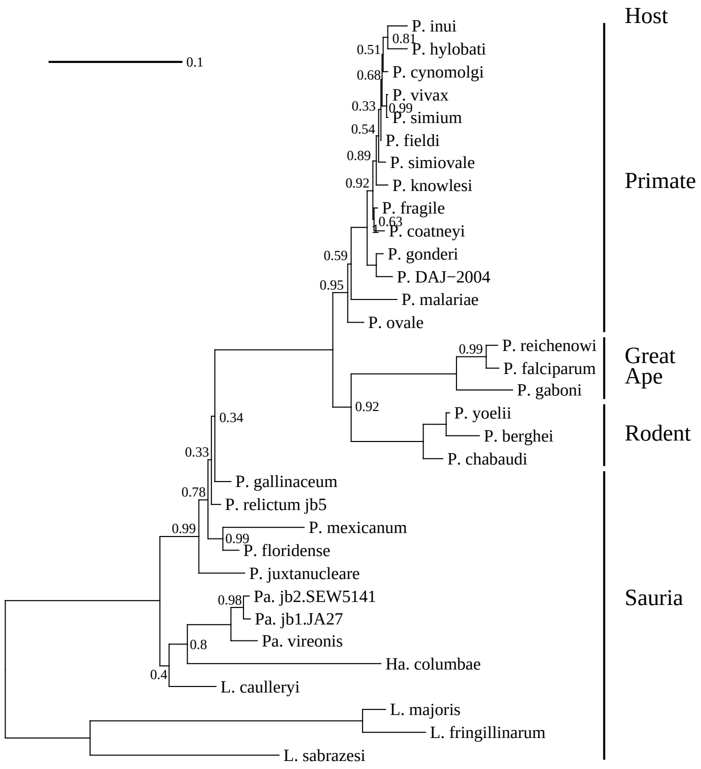

Supplementary Figure S2: **Phylogenetic tree of first and second codon position analyzed under $CAT + GTR + \Gamma_4$.** Bayesian phylogenetic reconstruction using PhyloBayes 3.0 [43]. The $CAT + GTR_{nt} + \Gamma_4$ substitution model was applied to first and second codon positions of the 33 taxa data set. *P. falciparum* and 2 of its relatives infecting great ape hosts, *P. reichenowi* and *P. gaboni*, formed a monophyletic clade with 3 rodent parasites, *P. yoelii*, *P. berghei* and *P. chabaudi* (posterior probability $PP = 0.92$). Posterior probabilities equal to 1 were removed.
