# Supplementary material for: Mitochondrial genes support a common origin of rodent malaria parasites and Plasmodium falciparum's relatives infecting great apes
Source: BMC Evol Biol. 2011 Mar 15;11:70. doi: 10.1186/1471-2148-11-70 (PMC3070646; doi:10.1186/1471-2148-11-70)
Supplement: Additional file 7 — Supplementary Table S5, Robustness of the support to the removal and addition of taxa. All codon positions were analyzed under GTR + Γ4 and GTR + Γ4 + I models, for Bayesian and ML methods, respectively. Addition or removal of taxa to the complete nucleotide data set comprising 33 taxa and 3 concatenated genes, 3308 sites. Phylogenetic analyses were performed under models GTRnt + Γ4 + I and GTRnt + Γ4, for maximum-likelihood (ML) and Bayesian (BI) methods, respectively. Cells display support as [PP, SH, BS], with PP: posterior probability (BI), SH: Shimodaira-Hasegawa-like support (ML), and BS: bootstrap support (ML). "*": not applicable. (a): PP and BS are summed over various positions of Hepatocystis species. Main lineages of mammal parasites are defined according to their host preference: "Rodent", "Primate" and "Great Ape" (see Additional file 1 and 8, Tables S1 and S6). "-" removal of species. "+" addition of species. "P. fal.": P. falciparum; "P. rei.": P. reichenowi; "P. gab.": P. gaboni; "P. yoe.": P. yoelii; "P. ber.": P. berghei; "P. cha.": P. chabaudi; "Hum": human primate parasites P. malariae and P. ovale; "Afr.": African primate parasites P. gonderi and P. DAJ-2004; "Asi": 10 Asian primate parasites; "Pla.": Plasmodium species infecting saurian hosts; "Hae.": Haemoproteus and Parahaemoproteus species; "Leu.": Leucocytozoon species; "Haemo." Haemosporidian species. [file 1471-2148-11-70-S7.PDF]

Sampled Group	Sampled Taxa	Support for Great Ape parasites sister group of:		
		Rodent	Primate+ Rodent	Primate
		PP, SH, BS	PP, SH, BS	PP, SH, BS
Great Ape Parasites	*-P. gab. -P. rei.*	0.999, 0.93, 0.854	0.001, * , 0.146	0.000, * , 0.000
	-P. fal. -P. rei.	0.997, 0.86, 0.771	0.002, * , 0.098	0.001, * , 0.094
	-P. fal. -P. gab.	0.926, 0.78, 0.676	0.074, * , 0.321	0.000, * , 0.003
	-P. rei.	0.999, 0.93, 0.884	0.001, * , 0.115	0.000, * , 0.001
	-P. gab.	0.996, 0.90, 0.785	0.004, * , 0.215	0.000, * , 0.000
	-P. fal.	0.997, 0.91, 0.792	0.003, * , 0.203	0.000, * , 0.005
	+8	0.995, 0.91, 0.828	0.005, * , 0.163	0.000, * , 0.009
Rodent Parasites	*-P. cha. -P. yoe.*	0.556, * , 0.519	0.444, 0.05, 0.475	0.000, * , 0.005
	-P. ber. -P. yoe.	1.000, 0.96, 0.911	0.000, * , 0.088	0.000, * , 0.001
	-P. ber. -P. cha.	0.991, 0.75, 0.779	0.009, * , 0.212	0.000, * , 0.008
	-P. yoe.	0.993, 0.90, 0.763	0.007, * , 0.235	0.000, * , 0.002
	-P. cha.	0.982, 0.67, 0.740	0.018, * , 0.251	0.000, * , 0.008
	-P. ber.	0.999, 0.93, 0.869	0.001, * , 0.127	0.000, * , 0.004
	+10	0.990, 0.77, 0.780	0.009, * , 0.206	0.000, * , 0.014
Primate Parasites	-2 Hum. -10 Asi.	0.149, * , 0.502	0.851, 0.26, 0.481	0.000, * , 0.017
	-2 Afr. -10 Asi.	0.985, 0.72, 0.745	0.015, * , 0.235	0.000, * , 0.020
	-2 Hum. -2 Afr.	0.967, 0.82, 0.708	0.032, * , 0.288	0.000, * , 0.004
	-10 Asi.	0.995, 0.79, 0.785	0.005, * , 0.212	0.000, * , 0.003
	-2 Hum.	0.959, 0.60, 0.732	0.040, * , 0.266	0.000, * , 0.002
	-2 Afr.	1.000, 0.94, 0.862	0.000, * , 0.131	0.000, * , 0.007
Sauria Parasites	-4 Leu. -5 Pla.	0.998, 0.89, 0.874	0.002, * , 0.117	0.000, * , 0.009
	-4 Hae. -5 Pla.	0.997, 0.89, 0.852	0.001, * , 0.037	0.002, * , 0.111
	-4 Hae. -4 Leu.	0.978, 0.64, 0.744	0.021, * , 0.251	0.000, * , 0.004
	-5 Pla.	1.000, 0.91, 0.879	0.000, * , 0.068	0.000, * , 0.053
	-4 Leu.	0.998, 0.77, 0.790	0.002, * , 0.207	0.000, * , 0.003
	-4 Hae.	0.998, 0.93, 0.868	0.002, * , 0.130	0.000, * , 0.002
	+27	0.996, 0.80, 0.784	0.004, * , 0.189	0.000, * , 0.018
+6 *Hepatocystis*[a]		0.998, 0.05, 0.875	0.001, * , 0.109	0.000, * , 0.016
+8, +10, +27 Pla.		0.987, 0.86, 0.763	0.011, * , 0.214	0.001, * , 0.017
+8, +10, +27, +6 Haemo.[a]		0.994, 0.00, 0.755	0.005, * , 0.216	0.000, * , 0.010

Supplementary Table S5: **Robustness of the support to the removal and addition of taxa.** All codon positions were analyzed under $GTR + \Gamma_4$ and $GTR + \Gamma_4 + I$ models, for Bayesian and ML methods, respectively. Addition or removal of taxa to the complete nucleotide data-set comprising 33 taxa and 3 concatenated genes, 3308 sites. Phylogenetic analyses were performed under models $GTR_{nt} + \Gamma_4 + I$ and $GTR_{nt} + \Gamma_4$, for maximum likelihood (ML) and Bayesian (BI) methods, respectively. Cells display support as [PP, SH, BS], with PP: posterior probability (BI), SH: Shimodaira-Hasegawa-like support (ML), and BS: bootstrap support (ML). "*": not applicable. (^{a}), PP and BS are summed over various positions of Hepatocystis species.: Main lineages of mammal parasites are defined according to their host preference: "Rodent", "Primate" and "Great Ape" (see Additional file 1 and 8, Tables S1 and S6). "-" removal of species. "+" addition of species. "*P. fal.*": *P. falciparum*; "*P. rei.*": *P. reichenowi*; "*P. gab.*": *P. gaboni*; "*P. yoe.*": *P. yoelii*; "*P. ber.*": *P. berghei*; "*P. cha.*": *P. chabaudi*; "Hum": human primate parasites *P. malariae* and *P. ovale*; "Afr.": African primate parasites *P. gonderi* and *P. DAJ-2004*; "Asi": 10 Asian primate parasites; "Pla.": *Plasmodium* species infecting saurian hosts; "Hae.": *Haemoproteus* and *Parahaemoproteus* species; "Leu.": *Leucocytozoon* species; "Haemo." *Haemosporidian* species.
