# Supplementary material for: Mitochondrial genes support a common origin of rodent malaria parasites and Plasmodium falciparum's relatives infecting great apes
Source: BMC Evol Biol. 2011 Mar 15;11:70. doi: 10.1186/1471-2148-11-70 (PMC3070646; doi:10.1186/1471-2148-11-70)
Supplement: Additional file 8 — Supplementary Table S6, Additional mitochondrial genes. Accession numbers of 51, 41 and 1 additional CytB, Cox1 and Cox3 genes, respectively, and parasite and host names (1 partial CytB genes). References: (a) Perkins and Schall (2002) [6]; (b) Perkins et al. (2007) [37]; (c) Cheesman et al. (2009) [76]; (d) Hall et al. (2005) [77]; (e) Escalante et al. (1998) [21]; (f) Seethamchai et al. (2008) [78]; (g) Martinsen et al. (2008) [36]; (h) Martinsen et al. (2007) [79]; (i) CytB + Cox1 + Cox3, Perkins (2008) [23]; (j) Rich et al. (2009) [26]. Abbreviation: "P.": Plasmodium species, "He.": Hepatocystis species. [file 1471-2148-11-70-S8.PDF]

Parasites	CytB	Cox1	Hosts	
P. reichenowi Bana([1])	EU560453 ([j])	-	*Pan troglodytes*	Great Ape
P. reichenowi Nino([1])	EU560454 ([j])	-	*Pan troglodytes*	
P. reichenowi Max([1])	EU560455 ([j])	-	*Pan troglodytes*	
P. reichenowi Rafiki1([1])	EU560456 ([j])	-	*Pan troglodytes*	
P. reichenowi Dibamba([1])	EU560457 ([j])	-	*Pan troglodytes*	
P. reichenowi Rafiki2([1])	EU560458 ([j])	-	*Pan troglodytes*	
P. reichenowi Loukoum([1])	EU560459 ([j])	-	*Pan troglodytes*	
P. reichenowi Gabon([1])	EU560466 ([j])	-	*Pan troglodytes*	
P. atheruri	AY099054 ([a])	DQ414588 ([b])	*Atherurus africanus*	Rodent
P. chabaudi adami	AB379670 ([c])	DQ414591 ([b])	*Thamnomys rutilans*	
P. chabaudi chabaudi	DQ414649 ([b])	XM_740254 ([d])	*Thamnomys rutilans*	
P. vinckei	DQ414650 ([b])	DQ414595 ([b])	*Grammomys surdaster*	
P. vinckei vinckei	DQ414651 ([b])	DQ414596 ([b])	*Grammomys surdaster*	
P. vinckei brucechwatti	DQ414652 ([b])	DQ414597 ([b])	*Praomys tullbergi*	
P. vinckei lentum	DQ414653 ([b])	DQ414598 ([b])	*Thamnomys rutilans*	
P. vinckei petteri	DQ414655 ([b])	DQ414600 ([b])	*Thamnomys rutilans*	
P. yoelii killicki	DQ414658 ([b])	DQ414603 ([b])	*Thamnomys rutilans*	
P. yoelii nigeriensis	DQ414659 ([b])	DQ414604 ([b])	*Thamnomys rutilans*	
He. sp.	AF069626 ([e])	-	*Papio nubensis*	Primate
He. sp. MFRC11	EU400408 ([f])	-	*Macaca fascicularis*	
He. sp. LDFB([1])	EU254526 ([g])	EU254569 ([g])	*Cynopterus brachyotis*	Bat
He. sp. MB3([1])	EU254528 ([g])	EU254571 ([g])	*Nanonycteris veldkampii*	
He. sp. MB6([1])	EU254527 ([g])	EU254570 ([g])	*Nanonycteris veldkampii*	
He. sp.	FJ168565 ([i])	FJ168565 ([i])	*Pteropus hypomelanus*	
P. azurophilum R([1])	EU254532 ([g])	EU254575 ([g])	*Anolis oculatus*	Lizard
P. azurophilum W([1])	EU254533 ([g])	EU254576 ([g])	*Anolis oculatus*	
P. sp. br67([1])	EU254537 ([g])	EU254580 ([g])	*Ameiva ameiva*	
P. sp. circ([1])	EU254531 ([g])	EU254574 ([g])	*Egernia stokesii*	
P. (Bennettinia) sp. PJ4([1])	EF011198 ([h])	EF011231 ([h])	*Gallus gallus*	Bird
P. (Giovannolaia) sp. 1536([1])	EF011187 ([h])	EF011220 ([h])	*Seiurus noveboracensis*	
P. (Giovannolaia) sp. 1542([1])	EF011188 ([h])	EF011221 ([h])	*Turdus migratorius*	
P. (Haemamoeba) sp. 594([1])	EF011176 ([h])	EF011209 ([h])	*Spizella passerina*	
P. (Haemamoeba) sp. P121([1])	EF011194 ([h])	EF011227 ([h])	*Emberiza hortulana*	
P. (Haemamoeba) sp. 805([1])	EF011179 ([h])	EF011212 ([h])	*Zonotrichia albicollis*	
P. (Haemamoeba) sp. 891([1])	EF011180 ([h])	EF011213 ([h])	*Dendroica coronata*	
P. (Huffia) sp. 182([1])	EF011168 ([h])	EF011201 ([h])	*Melospiza melodia*	
P. (Novyella) sp. 318([1])	EF011171 ([h])	EF011204 ([h])	*Agelaius phoeniceus*	
P. (Novyella) sp. 513([1])	EF011173 ([h])	EF011206 ([h])	*Seiurus aurocapilla*	
P. (Novyella) sp. 608([1])	EF011177 ([h])	EF011210 ([h])	*Turdus migratorius*	
P. relictum P113([1])	EF011193 ([h])	EF011226 ([h])	*Emberiza hortulana*	
P. relictum B170([1])	EU254538 ([g])	EU254581 ([g])	*Sialia mexicana*	
P. relictum 1([1])	EU254536 ([g])	EU254579 ([g])	*Zenaida macroura*	
P. sp. 1271([1])	EU254544 ([g])	EU254587 ([g])	*Hylocichla mustelina*	
P. sp. 1393([1])	EU254539 ([g])	EU254582 ([g])	*Accipiter striatus*	
P. sp. 1937([1])	EU254545 ([g])	EU254588 ([g])	*Turdus migratorius*	
P. sp. 2375([1])	EU254543 ([g])	EU254586 ([g])	*Aegolius acadicus*	
P. sp. Inca([1])	EU254547 ([g])	EU254590 ([g])	*Larosterna inca*	
P. sp. myna([1])	EU254542 ([g])	EU254585 ([g])	*Acridotheres tristis*	
P. sp. P159([1])	EU254541 ([g])	EU254584 ([g])	*Ixobrychus minutus*	
P. sp. P164([1])	EU254546 ([g])	EU254589 ([g])	*Anthus trivialis*	
P. sp. P166([1])	EU254540 ([g])	EU254583 ([g])	*Luscinia svecica*	

Supplementary Table S6: **Additional mitochondrial genes.** Accession numbers of 51, 41 and 1 additional CytB, Cox1 and Cox3 genes, respectively, and parasite and host names ([1] partial CytB genes). References: ([a]) Perkins and Schall (2002) [6]; ([b]) Perkins et al. (2007) [37]; ([c]) Cheesman et al. (2009) [76]; ([d]) Hall et al. (2005) [77]; ([e]) Escalante et al. (1998) [21]; ([f]) Seethamchai et al. (2008) [78]; ([g]) Martinsen et al. (2008) [36]; ([h]) Martinsen et al. (2007) [79]; ([i]) CytB + Cox1 + Cox3, Perkins (2008) [23]; ([j]) Rich et al. (2009) [26]. Abbreviation: "P.": *Plasmodium* species, "He.": *Hepatocystis* species.
