# Supplementary material for: Mitochondrial genes support a common origin of rodent malaria parasites and Plasmodium falciparum's relatives infecting great apes
Source: BMC Evol Biol. 2011 Mar 15;11:70. doi: 10.1186/1471-2148-11-70 (PMC3070646; doi:10.1186/1471-2148-11-70)
Supplement: Additional file 9 — Supplementary Table S7, Accession numbers of ClpC and ASL genes. Accession numbers of 27 ClpC and 18 ASL genes. (a): Taxa used for the estimation of the length of the 7-taxa tree. [file 1471-2148-11-70-S9.PDF]

Parasites	ClpC	ASL	Hosts
P. falciparum([a])	X87631	XM_001349541	Great Ape
P. berghei([a])	AF348337	AF262049	
P. chabaudi([a])	AY634622	XM_738483	Rodent
P. yoelii yoelii([a])	AY634624	XM_721443	
P. vivax([a])	AF348344	AF262051	
P. knowlesi([a])	AF348341	AF262052	
P. cynomolgi	AB471873	AF262054	
P. fieldi	AB471875	-	
P. simiovale	AB471881	-	
P. inui	AB471879	-	
P. hylobati	AB471878	-	Primate
P. fragile	AB471876	-	
P. coatneyi	AB471872	EU254704	
P. gonderi	AB471877	-	
P. ovale	AY634623	-	
P. malariae	AF348342	-	
P. gallinaceum([a])	AF348340	AF262053	
P. mexicanum	EU254619	EU254674	
P. relictum	EU254626	EU254682	
P. floridense	EU254620	EU254675	
P. juxtanucleare	AB435378	-	
Ha. syrnii	EU254643	-	
Ha. sp. 1774	EU254660	-	
Ha. sp. LV13Ecu	FJ467607	-	Sauria
Ha. columbae	EU254652	EU254702	
Ha. belopolskyi	-	EU254710	
Ha. fringillae	-	EU254711	
L. sp. 2208	EU254611	EU254665	
L. sp. 2109	EU254609	EU254663	
L. sp. P157	-	EU254664	

Supplementary Table S7: **Accession numbers of ClpC and ASL genes.** Accession number of 27 ClpC and 18 ASL genes. ([a]): Taxa used for the estimation of the length of the 7-taxa tree.
