# Supplementary material for: Mitochondrial genes support a common origin of rodent malaria parasites and Plasmodium falciparum's relatives infecting great apes
Source: BMC Evol Biol. 2011 Mar 15;11:70. doi: 10.1186/1471-2148-11-70 (PMC3070646; doi:10.1186/1471-2148-11-70)
Supplement: Additional file 10 — Supplementary Figure S3, Phylogenetic tree of 18 ASL genes. Bayesian reconstruction under the GTRnt + Γ4 model. Edges with PP < 0.9 were collapsed, and PP = 1 are not shown. The ASL phylogeny is not congruent with a monophyly of mammal malaria parasites. [file 1471-2148-11-70-S10.PDF]

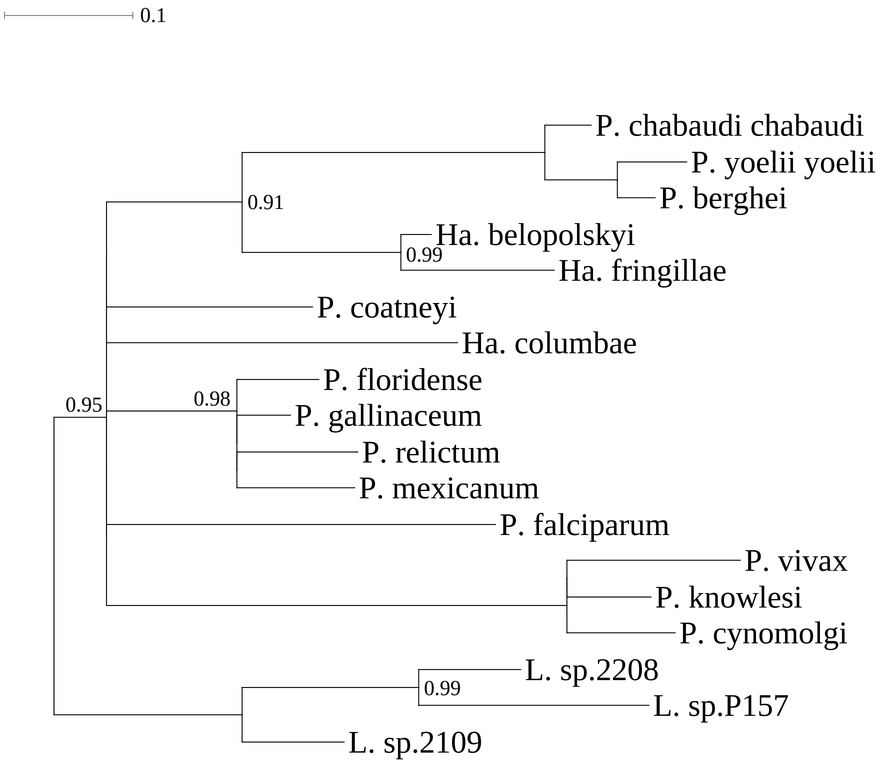

Supplementary Figure S3: **Phylogenetic tree of 18 ASL genes.** Bayesian reconstruction under the $GTR_{nt} + \Gamma_4$ model. Edges with $PP < 0.9$ were collapsed, and $PP = 1$ are not shown. The ASL phylogeny is not congruent with a monophyly of mammal malaria parasites.
