# Supplementary material for: Mitochondrial genes support a common origin of rodent malaria parasites and Plasmodium falciparum's relatives infecting great apes
Source: BMC Evol Biol. 2011 Mar 15;11:70. doi: 10.1186/1471-2148-11-70 (PMC3070646; doi:10.1186/1471-2148-11-70)
Supplement: Additional file 12 — Supplementary Table S8, Robustness of clade support in trees designed to display long branches. Each of the 9 taxon samples comprised the three most distantly related Leucocytozoon species, the 14 primate parasites, and two single representatives of parasites of great apes and of rodents, respectively. In each case, 14 taxa were removed from the complete nucleotide data-set comprising 33 taxa and 3 concatenated genes, 3308 sites. Phylogenetic analyses were performed under models GTRnt + Γ4 + I and GTRnt + Γ4, for maximum likelihood (ML) and Bayesian (BI) methods, respectively. Cells display support as follows: [PP, SH, BS], with PP: posterior probability (BI), SH: Shimodaira-Hasegawa-like support ("*": not applicable, ML), and BS: bootstrap support (ML). Main lineages of mammal parasites are defined according to their host preference: "Rodent", "Primate" and "Great Ape" (see Additional file 1, Table S1). "P. fal.": P. falciparum; "P. rei.": P. reichenowi; "P. gab.": P. gaboni; "P. yoe.": P. yoelii; "P. ber.": P. berghei; "P. cha.": P. chabaudi. [file 1471-2148-11-70-S12.PDF]

Sampled Taxa	Support for Great Ape parasites sister group of: Rodent		Primate+ Rodent		Primate	
	PP, SH, BS		PP, SH, BS		PP, SH, BS	
P. fal. P. ber.	0.007, * , 0.149		0.991, 0.86, 0.823		0.001, * , 0.028	
P. fal. P. cha.	0.525, 0.13, 0.508		0.466, * , 0.429		0.009, * , 0.063	
P. fal. P. yoe.	0.065, * , 0.285		0.922, 0.49, 0.601		0.013, * , 0.114	
P. gab. P. ber.	0.153, * , 0.198		0.445, * , 0.393		0.402, 0.01, 0.385	
P. gab. P. cha.	0.601, 0.13, 0.498		0.029, * , 0.076		0.369, * , 0.423	
P. gab. P. yoe.	0.115, * , 0.226		0.101, * , 0.181		0.783, 0.54, 0.572	
P. rei. P. ber.	0.000, * , 0.076		0.999, 0.94, 0.894		0.001, * , 0.029	
P. rei. P. cha.	0.193, * , 0.386		0.798, 0.27, 0.557		0.008, * , 0.057	
P. rei. P. yoe.	0.010, * , 0.151		0.984, 0.77, 0.745		0.005, * , 0.104	

Supplementary Table S8: **Robustness of clade support in trees designed to display long branches.** Each of the 9 taxon samples comprised the three most distantly related *Leucocytozoon* species, the 14 primate parasites, and two single representatives of parasites of great apes and of rodents, respectively. In each case, 14 taxa were removed from the complete nucleotide data-set comprising 33 taxa and 3 concatenated genes, 3308 sites. Phylogenetic analyses were performed under models $GTR_{nt} + \Gamma_4 + I$ and $GTR_{nt} + \Gamma_4$, for maximum likelihood (ML) and Bayesian (BI) methods, respectively. Cells display support as follows: [PP, SH, BS], with PP: posterior probability (BI), SH: Shimodaira-Hasegawa-like support ("*": not applicable, ML), and BS: bootstrap support (ML). Main lineages of mammal parasites are defined according to their host preference: "Rodent", "Primate" and "Great Ape" (see Additional file 1, Table S1). "*P. fal.*": *P. falciparum*, "*P. rei.*": *P. reichenowi*, "*P. gab.*": *P. gaboni*, "*P. yoe.*": *P. yoelii*, "*P. ber.*": *P. berghei*, "*P. cha.*": *P. chabaudi*.
